# Age- and Sex-Specific Analysis of Stroke Hospitalization Rates, Risk Factors, and Outcomes From German Nationwide Data

**DOI:** 10.1161/STROKEAHA.123.046118

**Published:** 2024-08-15

**Authors:** Dearbhla M. Kelly, Christiane Engelbertz, Peter M. Rothwell, Christopher D. Anderson, Holger Reinecke, Jeanette Koeppe

**Affiliations:** Wolfson Centre for the Prevention of Stroke and Dementia, Nuffield Department of Clinical Neurosciences, John Radcliffe Hospital, University of Oxford, United Kingdom (D.M.K., P.M.R.).; Department of Cardiology I – Coronary and Peripheral Vascular Disease, Heart Failure, University Hospital Muenster, Germany (C.E., H.R.).; Institute of Biostatistics and Clinical Research, University of Muenster, Germany (J.K.).; Program in Medical and Population Genetics, Broad Institute of Harvard and the Massachusetts Institute of Technology, Boston (C.D.A.).; McCance Center for Brain Health, Massachusetts General Hospital, Boston (C.D.A.).; Department of Neurology, Brigham and Women’s Hospital, Boston, MA (C.D.A.).

**Keywords:** hemorrhagic stroke, ischemic stroke, risk factors, sex characteristics, stroke

## Abstract

**BACKGROUND:**

Significant age and sex differences have been reported at each stage of the stroke pathway, from risk factors to outcomes. However, there is some uncertainty in previous studies with regard to the role of potential confounders and selection bias. Therefore, using German nationwide administrative data, we aimed to determine the magnitude and direction of trends in age- or sex-specific differences with respect to admission rates, risk factors, and acute treatments of ischemic and hemorrhagic stroke.

**METHODS:**

We obtained and analyzed data from the Research Data Centres of the Federal Statistical Office for the years 2010 to 2020 with regard to all acute stroke hospitalizations, risk factors, treatments, and in-hospital mortality, stratified by sex and stroke subtype. This database provides a complete national-level census of stroke hospitalizations combined with population census counts. All hospitalized patients ≥15 years with an acute stroke (diagnosis code: I60-64) were included in the analysis.

**RESULTS:**

Over the 11-year study period, there were 3 375 157 stroke events; 51.2% (n=1 728 954) occurred in men. There were higher rates of stroke admissions in men compared with women for both ischemic (378.1 versus 346.7/100 000 population) and hemorrhagic subtypes (75.6 versus 65.5/100 000 population) across all age groups. The incidence of ischemic stroke admissions peaked in 2016 among women (354.0/100 000 population) and in 2017 among men (395.8/100 000 population), followed by a consistent decline from 2018 onward. There was a recent decline in hemorrhagic stroke admissions observed for both sexes, reaching its nadir in 2020 (68.9/100 000 for men; 59.5/100 000 for women). Female sex was associated with in-hospital mortality for both ischemic (adjusted odds ratio, 1.11 [1.09–1.12]; *P*<0.001) and hemorrhagic stroke (adjusted odds ratio, 1.18 [95% CI, 1.16–1.20]; *P*<0.001).

**CONCLUSIONS:**

Despite improvements in stroke prevention and treatment pathways in the past decade, sex-specific differences remain with regard to hospitalization rates, risk factors, and mortality. Better understanding the mechanisms for these differences may allow us to develop a sex-stratified approach to stroke care.

Stroke is a leading cause of disability and death for both men and women worldwide.^1^ However, significant age and sex differences have been reported at each stage of the disease pathway, from risk factors to outcomes, and have become an increasing focus of health services and research.^2,3^

There are female-specific risk factors for stroke, including hypertensive disorders of pregnancy, oral contraception, and hormonal therapy use,^4^ but some conventional risk factors such as hypertension, smoking, and atrial fibrillation (AF) have also been associated with increased risk of stroke in women compared with men.^5^ The association of some of these traditional risk factors with stroke risk also appears to vary substantially with age.^6^ Age and sex differences have also been described in stroke presentation with progressive or nonfluctuating deficits more frequent at younger ages and nonfocal symptoms reported more commonly in women.^7,8^ In addition, recent meta-analyses have suggested that women are less likely to be treated with intravenous thrombolysis compared with men and seem to have greater disability and poorer quality of life poststroke with worse mortality rates.^9–11^ Previous German data has also demonstrated that those aged over 80 years are also less likely to receive contemporary acute stroke treatments.^12^

However, there is some conflicting data regarding some of the epidemiological disparities, for example, whether stroke incidence trends over time differ by sex. In an analysis of the Greater Cincinnati/Northern Kentucky Stroke Study, decreases in overall and ischemic stroke rates between 1993 and 2010 were reported for men but not women.^13^ In contrast, similar rates of decline for both sexes were described in the Atherosclerosis Risk in Communities study through 2017.^14^ Some population-based studies report an increasing stroke admission rate in younger age groups,^15–17^ although a recent systematic review suggests that the literature is heterogeneous in this regard.^18^

In addition, although previous studies have shown discrepancies according to sex, there is some uncertainty with regard to the role of potential confounders such as women’s longer life expectancy.^19^ In an analysis of the Oxford Vascular Study, there was no evidence of a worse outcome of stroke in women after adjusting for age and premorbid disability.^20^ Previous studies are often limited by generalizability and potential selection bias depending on the study population. Knowledge gaps also exist with regard to how more recent temporal trends in stroke vary with age and sex, which is of particular interest given the changes in therapeutic options and guidelines in the past decade.

Using German nationwide administrative data from 2010 to 2020, we aimed to address several knowledge gaps related to stroke epidemiology, risk factors, treatments, and outcomes. Specifically, we sought to describe temporal trends along with age- and sex-specific differences in stroke hospitalization rates, risk factors, treatments, and in-hospital mortality over a decade-long period. The availability of a large, diverse, and complete data set allows for a very inclusive analysis of how stroke affects men and women differently in a real-world setting and contributes to understanding how stroke burden has evolved over time on a nationwide scale.

## METHODS

### Data Availability

The data utilized in this study cannot be made available in the article, the supplemental files, or in a public repository due to German data protection laws (Bundesdatenschutzgesetz). Generally, access to data of statutory health insurance funds for research purposes is possible only under the conditions defined in German Social Law (SGB V § 287). Requests for data access can be sent as a formal proposal specifying the recipient and purpose of the data transfer to the appropriate data protection agency. Access to the data used in this study can only be provided to external parties under the conditions of the cooperation contract of this research project and after written approval by the sickness fund.

### Data Source

The introduction of a diagnosis and procedure-related remuneration system (German Diagnosis Related Groups [G-DRG] system) for all somatic in-patient services in Germany in 2003 has led to a precise and comprehensive acquisition of defined cases of illness. Detailed mandatory coding guidelines were implemented to ensure uniform documentation and billing. Thereby, all hospitals are obligated by law to transfer patient data on diagnoses, comorbidities, medical services, or procedures and procedure-related complications to the Institute for the Hospital Remuneration System.

Analyses were performed on our behalf by the Research Data Centres of the Federal Statistical Office and the Statistical Offices of the Laender (Statistisches Bundesamt, DESTATIS; https://www.destatis.de) for the years 2010 to 2020 with respect to acute stroke hospitalizations, risk factors, treatments, complications, and in-hospital mortality, stratified by sex and stroke subtype (all-cause/ischemic/hemorrhagic). The database comprises all in-patient treatments in German hospitals aggregated on a case basis, except for treatments in psychiatric or psychosomatic units. We excluded medical care provided by office-based specialists with special admitting. Due to data privacy protection, all subgroups <6 cases were excluded from the analysis. Access to the data was by remote execution directly on the anonymized original data. A statistical analysis program written in SAS (SAS 9.2; SAS Institute Inc, Cary, NC) was executed by the Research Data Centre.

### Diagnoses and Procedure Codes

Diagnoses, patient characteristics, comorbidities, and stroke complications are uniformly coded according to the *International Statistical Classification of Diseases and Related Health Problems, Tenth Revision, German modification* (ICD-10-GM; see Supplemental Material; Table S1). Similarly, endovascular and surgical procedures are uniformly coded according to the German Operation and Procedure Classification (see Supplemental Material; Table S2). Coding guidelines and annual adaptation by the German Institute for Medical Documentation and Information (Cologne, Germany) ensure uniform documentation. All hospitalized patients ≥15 years with an acute stroke (diagnosis code: I60-64) as their principal diagnosis were included in the analysis. Stroke subtype classification (ischemic or hemorrhagic) corresponded with the *ICD-10-GM* codes (see Supplemental Material; Table S1). The risk factors coded are those present on admission and do not necessarily reflect past medical history.

### Cost Calculations

Patient-level in-hospital costing data were derived from the Federal Bureau of Statistics (DESTATIS) and was available for all patients included in the analysis.^21^ The costing in the German DRG system followed the principle of full cost accounting, including actual direct costs and hospitals’ overhead costs. Individual items of patient consumption (eg, drugs and devices) as well as patient-specific utilization factors for calculating medical and nursing staff costs were taken into consideration for a very detailed bottom-up approach to the cost calculation. The costing methodology must follow by law the mandatory guidelines of the German National DRG Costing Study. Thus, the collected data of about 200 nationwide hospitals is used annually to calculate the German DRG system, which then represents the basis for hospital reimbursement in each of about 1200 DRGs for the 2000 hospitals in entire Germany.

### Statistical Analysis

Frequencies are given as case numbers per 100 000 population based on the sex-specific German population ≥15 years of the respective year. Proportions of hospitalizations are case numbers per total in-hospital cases ≥15 years and respective to sex. We did not include 95% CIs since the data shown here are the absolute numbers of all strokes in Germany in a specific year. Since the values are exact as opposed to a point estimate, a CI is not necessary. Mortality displays the percentage of in-hospital deaths within a designated subgroup. The analysis comprises all in-patient–treated acute stroke cases ≥15 years in Germany and does not represent a subgroup or sample. Mann-Whitney *U* tests and χ^2^ tests were used to test the significance of differences between 2 groups for continuous and categorical variables, respectively. Multivariable logistic regression analysis for in-hospital mortality was performed to evaluate the associations of age, sex, and in-hospital death adjusted by the patient’s risk profile in a full model (including all patients) and for female and male sex separately. The final model adjusted for the following covariates: age, sex, hypertension, dyslipidemia, diabetes, obesity, dementia, smoking, previous stroke, AF/flutter, peripheral artery disease (PAD), cerebrovascular disease (prior ischemic or hemorrhagic stroke), congestive heart failure, chronic kidney disease, and cancer and year of hospitalization. To address different patients’ risk profiles between female and male sex, we evaluated the interaction of sex with all other variables in the respective full models. All *P* values are 2-sided, and *P*<0.05 was considered significant. Statistical analysis was performed using SAS software (SAS 9.3: SAS Institute Inc) and the Web-based statistics software VassarStats (http://vassarstats.net; R. Lowry).

### Ethics

The data presented here were examined as part of the GenderVasc research project. This project was approved by the Ethics Committee Westfalen-Lippe and the Medical Faculty of the University of Muenster (No. 2019-21-f-S). This article conforms to the Strengthening the Reporting of Observational Studies in Epidemiology reporting guideline.^22^

## RESULTS

During the period of 2010 to 2020, there were 3 375 157 hospitalizations in Germany with acute stroke, of which 2 825 435 were ischemic and 549 722 were hemorrhagic events (Table S3). Men had a higher rate of ischemic stroke admissions with 378.1 per 100 000 population compared with 346.7 per 100 000 for women. The hemorrhagic stroke hospitalization rate was also higher for men compared with women (75.6 versus 65.5 per 100 000 population, respectively). These trends were consistent across all age groups for both subtypes (15–44; 45–64; 65–75; >75 years; Table S4).

**Figure 1.**
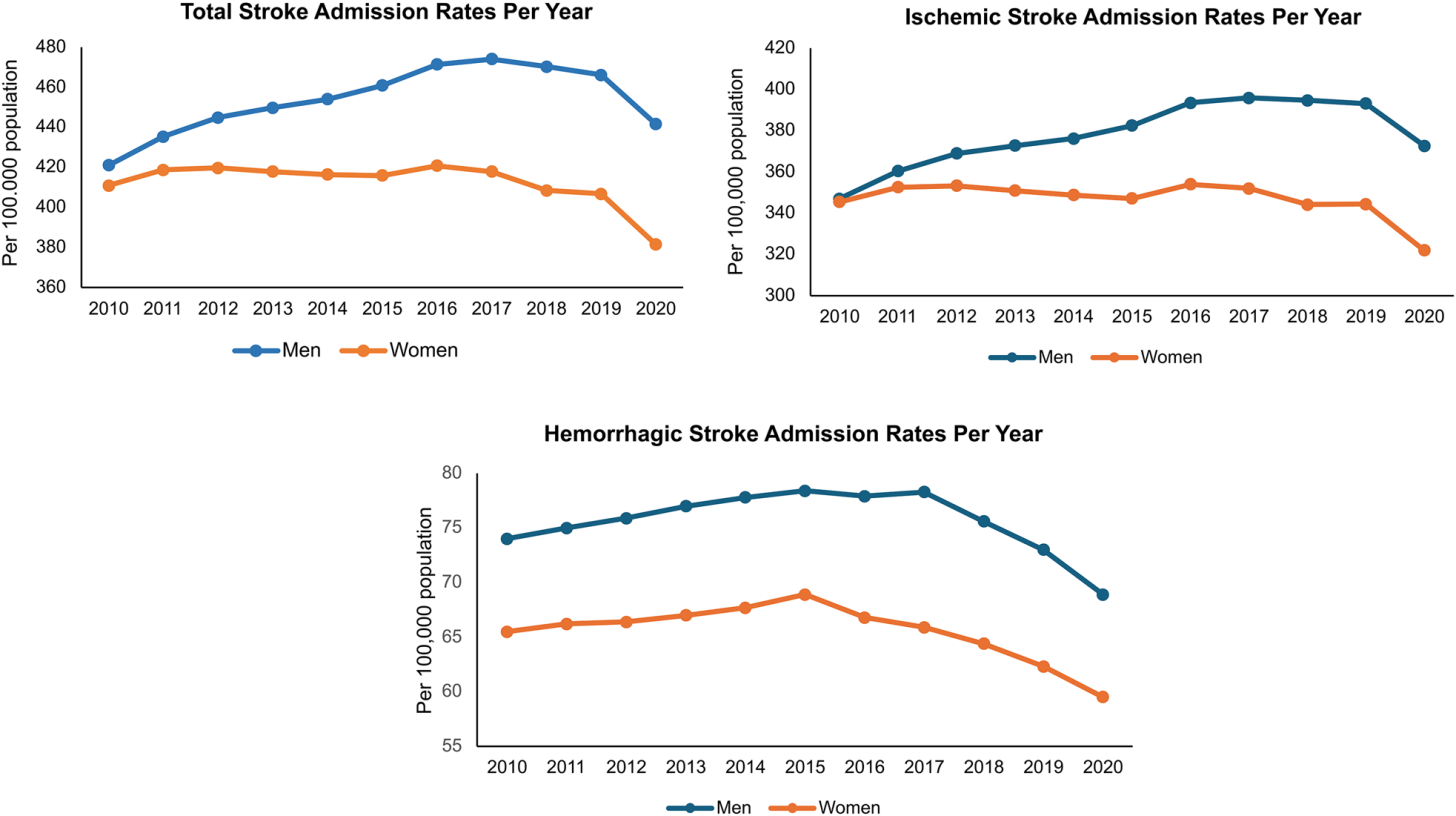
Acute stroke hospitalization rate (per 100 000 population) per year according to sex and subtype.

**Figure 2.**
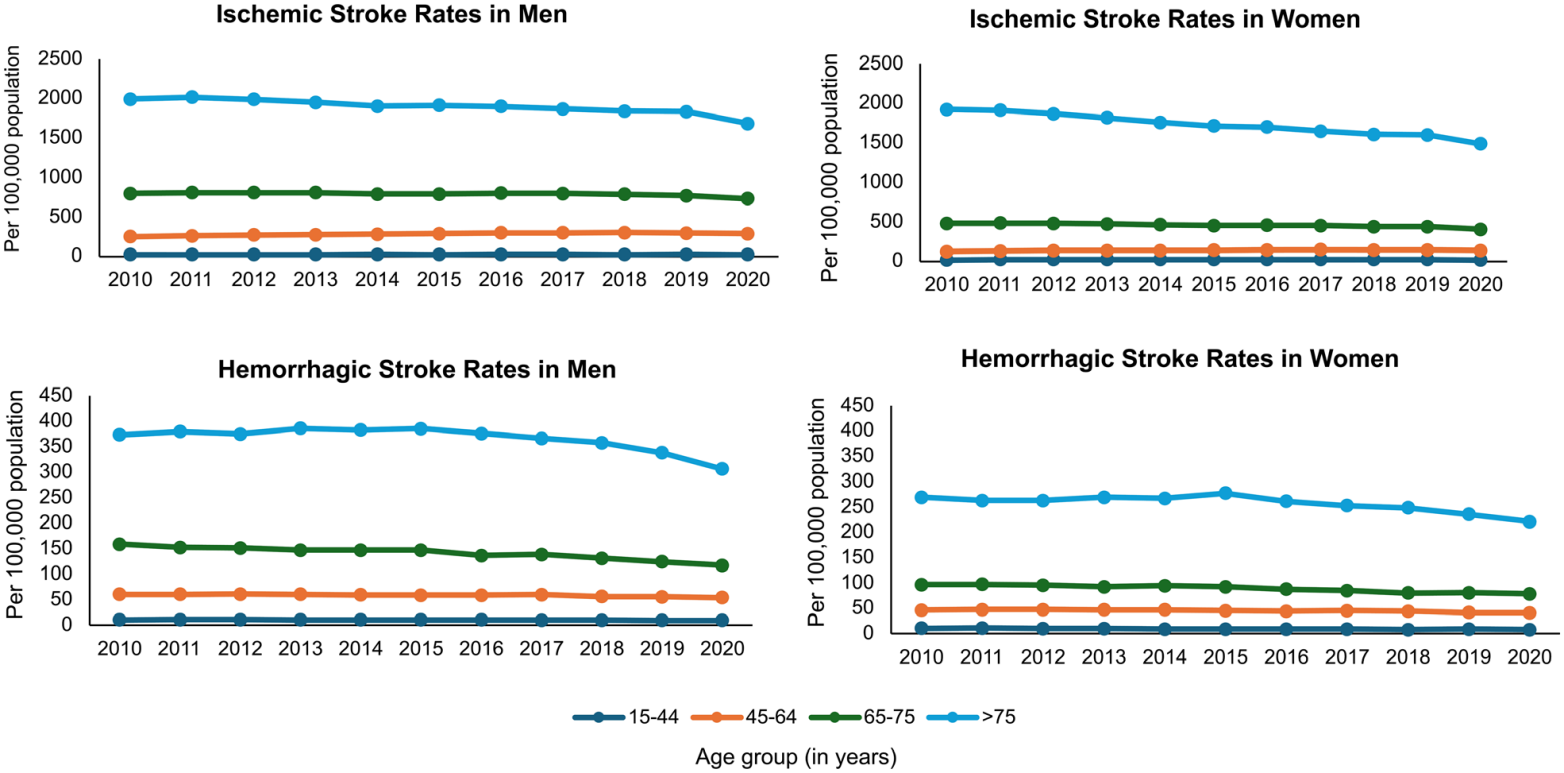
Acute stroke hospitalization rates per 100 000 population for ischemic and hemorrhagic strokes, by sex, age group, and time period.

Acute stroke admissions accounted for a similar percentage of all hospitalizations in Germany during this period for both sexes (Table S3). The total number of ischemic stroke admissions peaked in 2016 for women (354.0 per 100 000 population) and in 2017 for men (395.8 per 100 000 population) but then started to steadily fall for both sexes in 2018, particularly the ischemic stroke admissions for women (Table S3; Figure 1). There was also a recent decline in hemorrhage stroke admissions for both sexes with a nadir of 68.9 per 100 000 for men and 59.5 per 100 000 for women in 2020. In terms of age-specific stroke trends over this time period, the decline in ischemic and hemorrhagic stroke rates was most marked for those aged >75 years (Table S5; Figure 2).

There were significant sex differences in terms of baseline characteristics and risk factor profiles for ischemic stroke admissions (Table 1). Female patients were older (median age, 79 years; interquartile range, 71–85) and had a greater burden of AF (33.6 versus 25.2; *P*<0.001), hypertension (76.4% versus 74.3%; *P*<0.001), congestive heart failure (CHF; 12.5% versus 9.6%; *P*<0.001), chronic kidney disease (14.7% versus 12.3%; *P*<0.001), and dementia (6.9% versus 4.2%; *P*<0.001). These differences were largely driven by those aged >65 years (Table S6). In contrast, male patients were younger (median age, 73 years; interquartile range, 63–80) and had more cancer (3.2% versus 2.2%; *P*<0.001), coronary heart disease (18.5% versus 11.7%; *P*<0.001), diabetes (29.5% versus 26.8%; *P*<0.001), and a smoking history (6.2% versus 2.7%; *P*<0.001).

**Table 1.**
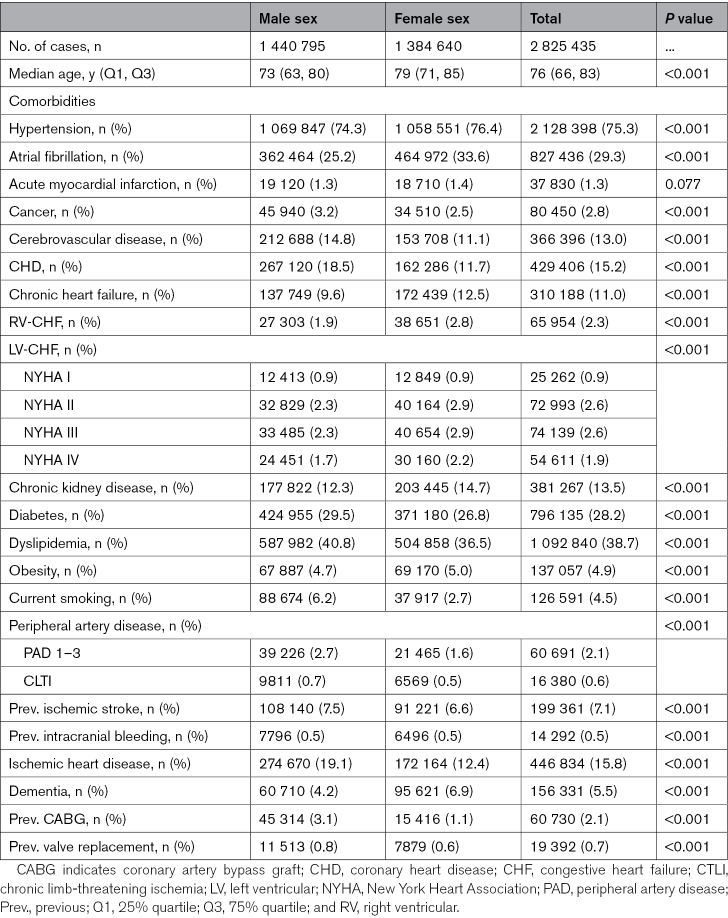
Baseline Characteristics of All Patients Hospitalized With Acute Ischemic Stroke in Germany (2010–2020) According to Sex

Similarly, there were also sex differences in the baseline characteristics of patients with hemorrhagic stroke (Table S7). Female patients were again older than their male counterparts (75 versus 72 years; *P*<0.001), but male patients had a higher prevalence of comorbidities, including hypertension (66.4% versus 64.2%; *P*<0.001), cancer (3.9% versus 2.8%; *P*<0.001), chronic kidney disease (9.5% versus 7.9%; *P*<0.001), and diabetes (19.1% versus 14.5%; *P*<0.001). These differences were consistent across all age categories (Table S8).

In terms of treatment of ischemic stroke, male patients underwent a greater number of carotid interventions (2.2% versus 1.1% and 1.2% versus 0.6% for endarterectomies and carotid stenting, respectively: both *P*<0.001; Table 2). Rates of intravenous thrombolysis were generally comparable between the sexes across all age categories, while rates of mechanical thrombectomies were higher in each female age category (Table S9). Complication rates were also broadly similar across the sexes, although male patients hospitalized with acute ischemic stroke experienced a slightly higher rate of complications, including a greater number of acute kidney injury (1.9% versus 1.7%; *P*<0.001) and sepsis (1.4% versus 1.0%; *P*<0.001) episodes, and an increased requirement for mechanical ventilation (5.2% versus 4.5%; *P*<0.001) with marginally higher associated health care costs (6649 versus 6235 euros; *P*<0.001). Among patients with hemorrhagic stroke, there were similar rates of craniectomy (1.9% for both) and intracerebral hemorrhage evacuation procedures (0.1% for both; Table S10). There were a higher number of bleeding events or need for blood transfusion among women (7.5% versus 6.6%; *P*<0.001) and a higher rate of acute kidney injury/need for renal replacement therapy (3.6% versus 2.1%; *P*<0.001) and sepsis (3.1% versus 1.9%; *P*<0.001) among men. These trends were consistent across all age categories (Table S11).

**Table 2.**
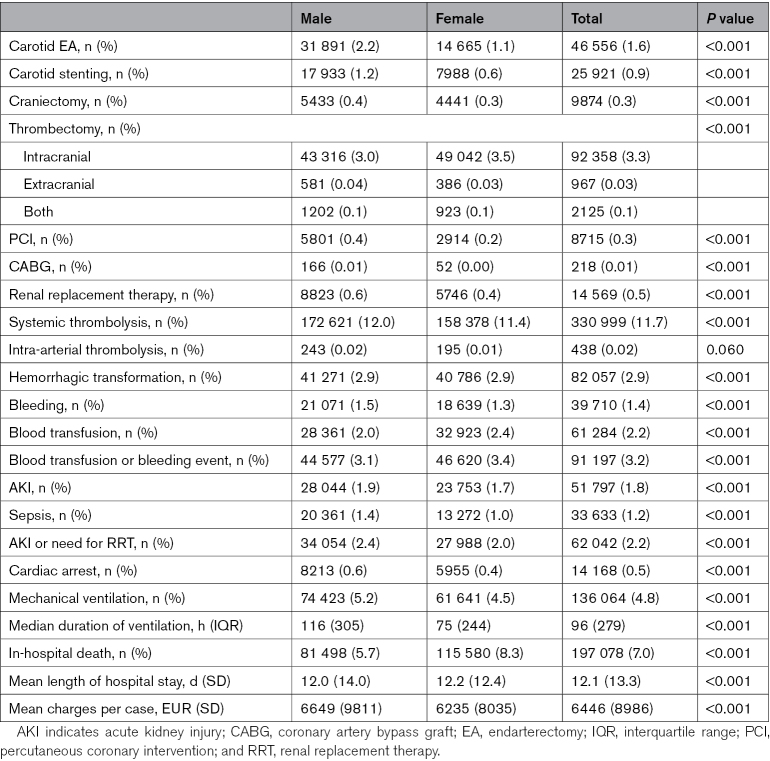
Acute Therapies and Complications Experienced by Patients Hospitalized With Acute Ischemic Stroke in Germany (2010–2020) According to Sex

**Figure 3.**
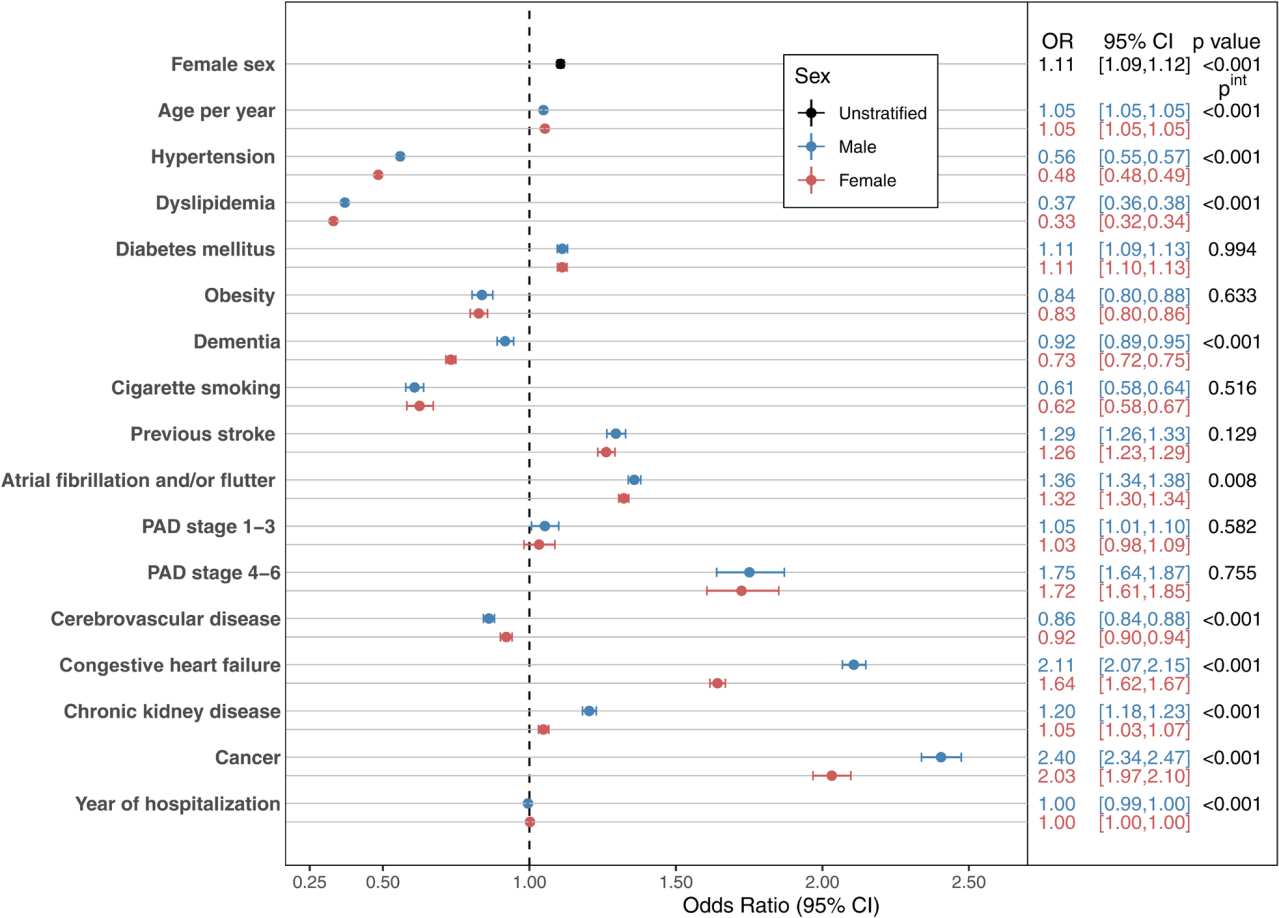
**Predictors of in-hospital mortality for all hospitalized patients with ischemic stroke (2010–2020) stratified by sex.** Multivariable adjusted ORs are presented with a final model including all other variables. OR indicates odds ratio; PAD, peripheral artery disease; and P^int^, interaction *P* value.

**Figure 4.**
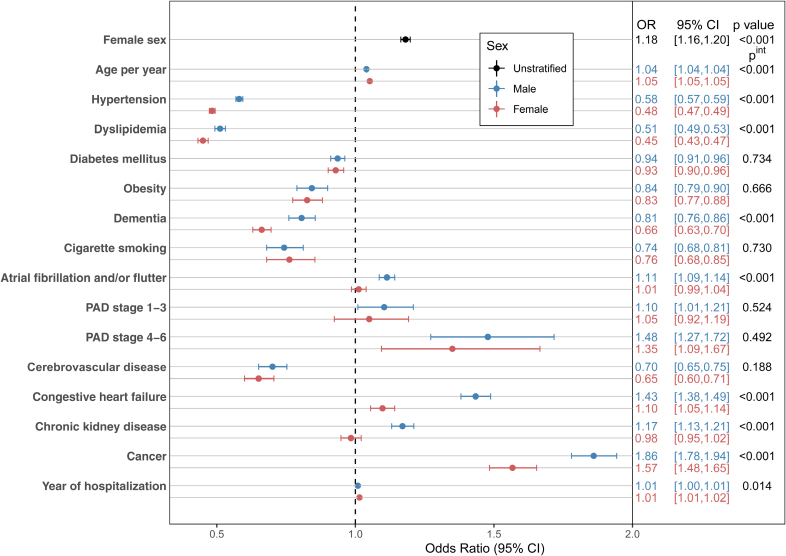
**Predictors of in-hospital mortality for all hospitalized patients with hemorrhagic stroke (2010–2020) stratified by sex.** Multivariable adjusted ORs are presented with a final model including all other variables. OR indicates odds ratio; PAD, peripheral artery disease; and P^int^, interaction *P* value.

The in-hospital mortality rate was higher among female patients across all age groups for both ischemic (8.3% versus 5.7%; *P*<0.001) and hemorrhagic stroke (19.9% versus 15.9%; *P*<0.001; Table 2; Tables S9 through S11). Figure 3 shows a forest-plot of a multivariable logistic regression analysis for predictors of in-hospital mortality for acute ischemic stroke patients stratified by sex. Female sex was independently predictive of in-hospital mortality after adjustment for potential confounders (adjusted odds ratio, 1.11 [95% CI, 1.09–1.12]; *P*<0.001). Increasing age, diabetes, previous stroke, AF, PAD, CHF, chronic kidney disease, and cancer were also independently predictive of in-hospital death for both sexes (Figure 3). The associations between advanced PAD, CHF, and cancer with in-hospital mortality were particularly strong (adjusted odds ratio, 1.75 [95% CI, 1.64–1.87]; 2.11 [95% CI, 2.07–2.15]; and 2.40 [95% CI, 2.34-2.47], respectively, for male patients with advanced PAD, CHF, and cancer; adjusted odds ratio, 1.72 [95% CI, 1.61–1.85]; 1.64 [95% CI, 1.62–1.67]; and 2.03 [95% CI, 1.97–2.10], respectively, for female patients with advanced PAD, CHF, and cancer). In the multivariable analysis, female sex was also independently predictive of in-hospital mortality for hemorrhagic stroke (adjusted odds ratio, 1.18 [95% CI, 1.16–1.20]; *P*<0.001; Figure 4). Similarly, advanced PAD, CHF, and cancer were most strongly associated with the risk of death for both sexes with hemorrhagic stroke.

## DISCUSSION

This comprehensive, nationwide study of sex-specific stroke epidemiology encompassed all hospitalized stroke cases from Germany between 2010 and 2020. During this decade-long period, there were consistently higher stroke hospitalization rates in men compared with women, with a decline in rates observed for both sexes in recent years. There were marked differences in risk factor profiles with broadly similar treatments received. Even after adjustment for potential confounders, female sex was independently associated with in-hospital mortality, though this association was stronger for hemorrhagic than ischemic stroke.

By analyzing data from 2010 to 2020, the study sheds light on temporal trends in stroke incidence rates, highlighting a decline in rates of hospitalized stroke for both sexes from 2018, particularly for those aged >75 years. While the COVID-19 epidemic could have influenced stroke presentations in 2020,^23^ it is important to note that the declining trend began before the year 2020 and our results are in keeping with reductions in stroke hospitalization and severity that have been observed in other developed countries during this timeframe.^24–26^ These changes may reflect improved control of cardiovascular risk factors, the availability of high-quality evidence and guidelines on best practices in primary and secondary stroke prevention,^27,28^ and more proactive implementation of stroke prevention clinics. It is worth noting that changes in admission rates do not necessarily parallel changes in severity. Germany has a health care system with a very high admittance rate for transient ischemic attack and minor stroke, and as such, hospitalization rates are a reasonable proxy for stroke incidence rates in this setting.

The higher admission rates observed in men in all age groups are consistent with most stroke incidence studies from developed countries^29,30^ as well as more recently published data from a population-based registry study in Northern France and the UK Biobank.^5,31^ This discrepancy may relate to the earlier onset of atherosclerosis in men^32^ and the initial protective effect of estrogen on cerebral circulation for women.^33^ Certain studies have also suggested that women are more likely to present with atypical, nonfocal stroke symptoms and to be misdiagnosed, which may confound some of these apparent differences.^34,35^

Unlike several previous studies, we did not find an increasing stroke admission rate in younger age groups (15–44 years) in either men or women.^15–17^ The increasing hospitalization rates in other nationally representative samples have been proposed to relate to the increasing prevalence of conventional cardiovascular risk factors, including hypertension, diabetes, and obesity, in younger people.^17^ However, it is likely that there are differences in risk profiles between and within countries. In particular, we were unable to study race or ethnicity in this nationwide sample as racial statistics are not routinely recorded in Germany. The incidence of stroke and prevalence of certain risk factors in young adults are higher among Black and Hispanics,^36^ and they may not be broadly represented in this population. The declining hospitalization rates in the >75-year age group may reflect the impact of improved risk factor control and prevention strategies that have been implemented in the last 2 decades.

There were important sex differences in the risk factor profiles between men and women, as women with ischemic stroke were older with more comorbid hypertension, CHF, chronic kidney disease, and dementia. Although we did not specifically look at the impact of comorbidity burden in this study, there is a pattern present suggestive of greater multimorbidity and frailty in female patients with acute stroke, and further research is needed to determine to what extent the number of comorbidities or certain specific disease clusters may contribute to the excess mortality observed in female patients.^37^ Understanding these differences can help tailor preventive strategies to specific patient groups.

In this study, we examined acute and hyperacute treatment patterns for both sexes. This information is critical for assessing whether there are disparities in access to acute stroke treatments. Although some sex differences were observed in terms of therapies and complications, overall, there were similar rates of intravenous thrombolysis and interventions. Our findings contrast with those of a recent meta-analysis of 17 observational studies that indicated that women had 13% lower odds of receiving intravenous thrombolysis treatment than men.^9^ However, this estimate was unadjusted, and there was considerable variability between the results of the individual studies included. Apparent discrepancies in carotid interventions and thrombectomy rates between men and women may reflect underlying differences in stroke etiology or subtype prevalence that would not have been captured in this *ICD-10*-coded data. Men tend to experience more large artery strokes due to carotid artery stenosis, while the incidence of cardio-embolic stroke secondary to AF is greater in women.^38^ The latter is more likely to lead to large vessel occlusion necessitating mechanical thrombectomy.^39^ AF is also a disease with an incidence strongly linked to age,^40^ and the women in this sample are older.

In contrast to some prior studies,^41,42^ female sex was independently associated with in-hospital mortality even after multivariable adjustment. Several factors may contribute to women’s excess risk of mortality, including advanced age and a greater burden of prestroke dementia and AF.^41^ Prestroke mild cognitive impairment and dementia have both been independently associated with poststroke mortality.^43^ It has also been shown that women with AF may be undertreated relative to men,^44^ and there may be a 20% higher risk of bleeding complications with oral anticoagulation in women compared with men.^45^ This increased bleeding risk may explain the particularly higher risk for hemorrhagic stroke mortality in women, which is consistent with the results from other large-scale epidemiological data sets.^10^ In addition, it has been found that women are less likely to get stroke code activation when presenting with intracerebral hemorrhage compared with men.^46^ Stroke severity and premorbid disability have been proposed to contribute to sex differences in stroke mortality,^41^ and as we were unable to adjust for these factors in this administrative data set, there may be residual confounding. However, recent studies have shown that there are sex-specific functional cerebral asymmetries that may help explain differences in acute stroke severity,^47^ and it would seem that long-term mortality remains higher in women at 1- and 5-years poststroke, suggesting that there may also be some underlying important biological differences or pathobiology.^41^ Compounding the problem, women remain underrepresented in stroke trials, so studies have not been designed to conclusively assess the sex-specific effects of experimental therapies and interventions.^48^ By identifying sex-specific differences in in-hospital mortality, this study contributes to the understanding of factors influencing poststroke outcomes and highlights the importance of considering sex when assessing stroke prognosis.

Our study has several strengths, including the presentation of high-quality, complete, and generalizable data from a large-sized, unselected entire population of a nation free of the limitations of convenience samples. The reliability and validity of the used *ICD* and German Operation and Procedure Classification codes are also very high because they directly impact the hospitals’ reimbursement. However, our study also had a few limitations. First, data acquisition was case-based rather than patient-based, and therefore, there could have led to a certain number of double-counted patients, for example, by hospital transfer, and we were therefore also unable to distinguish incidents from recurrent events. Second, detail of certain potential confounders, such as time to presentation or treatment, initial stroke severity, prior medications, and preexisting functional or socioeconomic status within which there may also have been sex differences that could have influenced the subsequent outcomes, was not available from this administrative data. Furthermore, in this context, there may have been a risk of misclassification bias due to coding errors, incomplete information, changes over time, heterogeneity, lack of specificity, or clinical validation. Future studies should also include data on stroke severity to provide a more nuanced understanding of the sex-specific differences in outcomes. Third, it may be difficult to interpret any apparent differences without more detailed information on underlying etiological stroke subtypes. Etiological classification of stroke into different causative subtypes, like the widely used TOAST (Trial of ORG 10172 in Acute Stroke Treatment) system,^49^ is important as different subtypes may be associated with different risk factors, management, and prognosis.^50,51^ Furthermore, we did not include the *ICD* coding for transient ischemic attack events, and thus milder cerebrovascular events may be underrepresented in this national study, which, on the contrary, resulted in a more homogenous cohort. Fourth, although we adjusted our analyses for several apriori-selected confounders, we cannot exclude the possibility of residual confounding. Fifth, the study focuses on in-hospital mortality and does not provide information on long-term outcomes, which are critical for understanding the full impact of stroke on patients’ lives. Sixth, the findings are based on German administrative data, and while the large sample size is a strength, the results may not be fully generalizable to other countries or health care systems with different demographic profiles or health care practices. Despite these limitations, administrative data are considered to be most appropriate to track trends in national estimates of stroke over multiple years.^52^ Efforts to improve the accuracy and completeness of coding in administrative data sets would enhance the reliability of future studies in this area.

## CONCLUSIONS

Sex differences in stroke risk, management, and outcome have become an increasing focus of health services and research as they have important clinical and policy implications. This study addresses several important knowledge gaps related to stroke, particularly with a focus on sex-specific differences in epidemiology and outcomes. It provides valuable insights that can guide future research and inform strategies for improving stroke care and reducing disparities in outcomes between men and women. Ongoing monitoring of sex differences in the burden of stroke from national or nationally representative data sets such as this one will be needed to determine if disease rates and outcomes among men and women continue to diverge. Additional research is required to elucidate why there is evidence for an independent association between female sex and in-hospital mortality poststroke, whether there is sex-specific pathophysiology, and what its relevance is to clinical practice. Investigating the role of additional confounders such as lifestyle factors, socioeconomic status, and access to health care could provide further insights into the observed sex differences. Expanding the analysis to include long-term outcomes such as functional status, quality of life, and recurrent stroke rates would provide a more complete picture of the impact of sex on stroke recovery. Comparing the findings with data from other countries or health care systems could help validate the results and explore the generalizability of the conclusions. There is a need for stroke studies to present sex-disaggregated analyses of risk factors, interventions, and outcomes to better understand these mechanisms and to ensure evidence-based health care for both men and women.

## ARTICLE INFORMATION

### Sources of Funding

This study was conducted within the framework of the GenderVasc project (Gender-specific real care situation of patients with arteriosclerotic cardiovascular diseases) funded by The Federal Joint Committee, Innovation Committee (G-BA, Innovationsfond, number 01VSF18051).

### Disclosures

Dr Reinecke reports personal fees from Daiichi, MedUpdate, DiaPlan, NeoVasc, NovoNordisk, StreamedUp, and Corvia; grants and personal fees from Pluristem; and grants from BMS/Pfizer, Bard, and Biotronik; all outside the submitted work. Dr Anderson reports grants from the US National Institutes of Health and the American Heart Association. Dr Engelbertz has received travel support from Abbott outside the submitted work. Dr Rothwell has in the past provided consultancy services for Abbott Vascular, Bayer, Bristol-Myers Squibb, and Sanofi US Services. Dr Koeppe has received research funding from Stryker, outside the submitted work. The other author reports no conflicts.

### Supplemental Material

Tables S1–S11

## Supplementary Material


