# Supplementary material for: Age- and Sex-Specific Analysis of Stroke Hospitalization Rates, Risk Factors, and Outcomes From German Nationwide Data
Source: Stroke. 2024 Aug 15;55(9):2284–94. doi: 10.1161/STROKEAHA.123.046118 (PMC11346720; doi:10.1161/STROKEAHA.123.046118)
Supplement: Supplementary file 1 [file str-55-2284-s001.pdf]

SUPPLEMENTAL MATERIAL

Age- and sex-specific analysis of stroke hospitalization rates, risk factors and outcomes from German nationwide data

Dearbhla M. Kelly, *MBBChBAO MSc DPhil MRCPI,* [1] Christiane Engelbertz, *PhD*, Peter Rothwell, *PhD FRCP FMedSci,*[1] Christopher D. Anderson, MD MSc,[4,5,6] Holger Reinecke, *MD,*[22] Jeanette Koeppe, *PhD.*[3]

[1]Wolfson Centre for the Prevention of Stroke and Dementia, Nuffield Department of Clinical Neurosciences, John Radcliffe Hospital, University of Oxford, United Kingdom.

[2]Department of Cardiology I – Coronary and Peripheral Vascular Disease, Heart Failure, University Hospital Muenster, Cardiol, Muenster, Germany.

[3]Institute of Biostatistics and Clinical Research, University of Muenster, Muenster, Germany.

[4]Program in Medical and Population Genetics, Broad Institute of Harvard and the Massachusetts Institute of Technology, Boston, MA, USA.

[5]McCance Center for Brain Health, Massachusetts General Hospital, Boston, MA, United States.

[6] Department of Neurology, Brigham and Women's Hospital, Boston, MA, United States.

Corresponding author:

Dr. Dearbhla Kelly

Wolfson Centre for the Prevention of Stroke and Dementia, Nuffield Department of Clinical Neurosciences, John Radcliffe Hospital, University of Oxford, United Kingdom.

Tel no: +353877552258

Email: dearbhla.kelly@gbhi.org

TABLE OF CONTENTS

Page Number

Supplementary Tables

Table S1: Diagnoses and Procedure Codes…………………….…………………...3

Table S2: Definitions of complications and procedures according to ICD-10-GM and OPS codes….……………………………………….……………………..4

Table S3: Absolute annual stroke admission rates and crude rates per 100,000 population according to sex………………………...……………………..6

Table S4: Age-specific annual stroke admission rates according to sex…..….……..7

Table S5: Hospitalization rates for ischaemic and haemorrhagic strokes, by sex, age group and time period…………………………………….……….8

Table S6: Baseline characteristics of patients hospitalized with acute ischaemic stroke in Germany (2010-2020), by sex and age group.…………….……...….……10

Table S7: Baseline characteristics of all patients hospitalized with acute haemorrhagic stroke in Germany (2010-2020) according to sex………………………13

Table S8: Baseline characteristics of patients hospitalized with acute haemorrhagic stroke in Germany, by sex and by time period………...……….…….14

Table S9: Acute therapies and complications experienced by patients hospitalized with acute ischaemic stroke in Germany (2010-2020) according to sex and age group……………………….………………...…….17

Table S10: Acute therapies and complications experienced by patients hospitalized with acute haemorrhagic stroke in Germany (2010-2020) according to sex…………………………………………….……..….………..21

Table S11: Acute therapies and complications experienced by patients hospitalized with acute haemorrhagic stroke in Germany (2010-2020) according to sex and age group………………………………...……….……..22

Table S1: Diagnoses and Procedure Codes

Diagnosis	ICD 10 – GM – 2020
**Cerebrovasular disease**	**I60 – I64**
Subarachnoid haemorrhage	I60.-
Intracerebral haemorrhage	I61.-
Other, non-traumatic intracerebral haemorrhage	I62.-
Cerebral infarction	I63.-
Stroke, not referred to as bleeding or infarction	I64.-
Secondary Diagnosis	
**Chronic Kidney Disease**	N18
**Diabetes Mellitus**	E10 -E11
**Obesity**	E66.-
**Dyslipidaemia**	E78.-
**Current Smoking Use**	F17
**Hypertensive Disease**	I10-I15
**Atrial Fibrillation**	I48.-
**Acute Myocardial Infarction**	I21.-, I22.-
**Coronary Heart Disease**	I25.1
**Congestive Heart Failure**	I50.-
**Left Ventricular Failure**	I50.1 et seqq.
**Right Ventricular Failure**	I50.0
**Peripheral Artery Disease**	I70.20-I70.25
**Dementia**	F00.-,F01.-
**Cancer**	C
**Previous ischaemic stroke**	I69.3, I69.4
**Previous intracranial bleeding**	I69.0, I69.1, I69.2
**Previous coronary artery bypass grafting**	Z95.1
**Previous valve replacement**	Z95.1

Acute stroke as principal diagnosis and co-morbidities as secondary diagnoses were coded according to the German Modification of the International Statistical Classification of Diseases and Related Health Problems 10th Revision (ICD-10-GM).

Table S2: Definitions of complications and procedures according to ICD-10-GM and OPS codes

Complication	Parameter / ICD-10-GM code / OPS code
Sepsis, unspecified	A41
Transfusion	8-800.c
Bleeding event	H44.8, K92, T81.0-3, K81.7
Haemorrhagic transformation	I60 – I62
Acute kidney injury	N17.-
Renal replacement therapy	8-853, 8-854, 8-855
Cardiac arrest	8-771
Coronary artery bypass graft	5-36 et seqq.
Percutaneous coronary intervention	8-837 et seqq.
Valve surgery	Z95.2
Systemic thrombolysis	8-020.8
Intra-arterial thrombolysis	8-020.d
Carotid endarterectomy	5-380.0, 5-380.1
Carotid stenting	8-840.0h, 8-840.0j, 8-840.0k, 8-840.0m, 8-840.1h, 8-840.1j, 8-840.1k, 8-840.1m, 8-840.2h, 8-840.2j, 8-840.2k, 8-840.2m, 8-840.3h, 8-840.3j, 8-840.3k, 8-840.3m
Carotid interposition graft	5-383.0
Craniectomy	5-010-10, 5-010-11, 5-010-12, 5-010-13, 5-010-14
ICH evacuation	5-014.1
Evacuation of extracranial haemorrhage	5-013.1
Extracranial thrombectomy	8-836.81
Intracranial thrombectomy	8-836.80

A code marked by a dash (-) indicates that all subheadings of this code are included.

ICH, intracerebral haemorrhage

Acute stroke as principal diagnosis and co-morbidities as secondary diagnoses were coded according to the German Modification of the International Statistical Classification of Diseases and Related Health Problems 10th Revision (ICD-10-GM). Procedures were coded according to the German Procedure Classification (OPS).

Table S3. Absolute annual stroke admission rates and crude rates per 100,000 population according to sex

		2010	2011	2012	2013	2014	2015	2016	2017	2018	2019	2020	
**Absolute numbers of stroke**													**Total**
**Ischaemic**	**Men**	119,722	121,453	125,064	127,087	129,240	133,544	137,797	138,924	138,756	138,311	130,897	1,440,795
	**Women**	125,423	126,382	126,983	126,427	126,112	126,242	129,054	128,515	125,804	126,026	117,672	1,384,640
	**Total**	245,145	247,835	252,047	253,514	255,352	259,786	266,851	267,439	264,560	264,337	248,569	2,825,435
**Haemorrhagic**	**Men**	25,523	25,281	25,734	26,267	26,740	27,369	27,277	27,470	26,600	25,679	24,219	288,159
	**Women**	23,794	23,733	23,873	24,142	24,466	25,049	24,345	24,070	23,536	22,805	21,750	261,563
	**Total**	49,317	49,014	49,607	50,409	51,206	52,418	51,622	51,540	50,136	48,484	45,969	549,722
**Overall**		294,462	296,849	301,654	303,923	306,558	312,204	318,473	318,979	314,696	312,821	294,538	3,375,157
**Stroke rates per 100,000 population**													
**Ischaemic**	**Men/100,000**	347.0	360.4	368.9	372.7	376.2	382.4	393.5	395.8	394.6	393.1	372.6	378.1
	**Women/100,000**	345.4	352.5	353.3	350.9	348.8	347.1	354.0	351.9	344.1	344.4	322.0	346.7
**Haemorrhagic**	**Men/100,000**	74.0	75.0	75.9	77.0	77.8	78.4	77.9	78.3	75.6	73.0	68.9	75.6
	**Women/100,000**	65.5	66.2	66.4	67.0	67.7	68.9	66.8	65.9	64.4	62.3	59.5	65.5
**Overall**		**415.8**	**426.8**	**431.9**	**433.4**	**434.8**	**437.9**	**445.6**	**445.4**	**438.7**	**435.8**	**410.9**	**432.5**
		**Percentage of stroke admissions from all hospitalisations**											
**Ischaemic**	**%Men/admissions**	1.55	1.53	1.55	1.56	1.55	1.58	1.61	1.63	1.63	1.62	1.75	1.59
	**%Women/admissions**	1.40	1.39	1.37	1.36	1.33	1.33	1.34	1.34	1.33	1.33	1.43	1.36
**Haemorrhagic**	**%Men/admissions**	0.33	0.32	0.32	0.32	0.32	0.32	0.32	0.32	0.31	0.30	0.32	0.32
	**%Women/admissions**	0.27	0.26	0.26	0.26	0.26	0.26	0.25	0.25	0.25	0.24	0.26	0.26

Table S4: Absolute age-specific stroke admission rates for 2010-2020 according to sex

	15-44 years	45-64 years	65-75 years	>75 years	Total
**Ischaemic stroke**					
**Men**	39,867 (2.8)	376,293 (26.1)	384,502 (26.7)	640,133 (44.4)	1,440,795
**Women**	33,981 (2.5)	183,345 (13.2)	251,094 (18.1)	916,220 (66.2)	1,384,640
**Total**	73,848 (2.6)	559,638 (19.8)	635,596 (22.5)	1,556,353 (55.1)	2,825,435
**Haemorrhagic stroke**					
**Men**	17,290 (6.0)	78,563 (27.3)	68,991 (23.9)	123,315 (42.8)	288,159
**Women**	15,181 (5.8)	60,742 (23.2)	49,233 (18.8)	136,407 (52.2)	261,563
**Total**	32,471 (5.9)	139,305 (25.3)	118,224 (21.5)	259,722 (47.2)	549,722
**All stroke**					
**Men**	57,157 (3.3)	454,856 (26.3)	453,493 (26.2)	763,448 (44.2)	1,728,954
**Women**	49,162 (3.0)	244,087 (14.8)	300,327 (18.2)	1,052,627 (63.9)	1,646,203
**Total**	106,319 (3.2)	698,943 (20.7)	753,820 (22.3)	1,816,075 (53.8)	3,375,157

The percentage refers to the total number of events in the respective sex group.

Table S5: Hospitalization rates per 100,000 population for ischaemic and haemorrhagic strokes, by sex, age group and time period

Age Groups (years)	Time period										
	Ischaemic										
**Men**	**2010**	**2011**	**2012**	**2013**	**2014**	**2015**	**2016**	**2017**	**2018**	**2019**	**2020**
**15-44**	23.3	24.1	24.3	23.9	24.7	23.9	25.1	24.6	23.9	24.6	23.8
**45-64**	251.0	260.0	271.6	272.8	280.2	286.7	297.4	297.5	300.3	293.3	285.6
**65-75**	794.6	805.5	805.4	805.3	787.6	789.3	798.8	796.2	784.8	769.2	731.0
**>75**	1,990.4	2,016.2	1,987.6	1,952.4	1,903.9	1,914.4	1,898.4	1,869.3	1,839.6	1,833.9	1,684.3
**Total**	347.0	360.4	368.9	372.7	376.2	382.4	393.5	395.8	394.6	393.1	372.6
**Women**	**Ischaemic**										
	**2010**	**2011**	**2012**	**2013**	**2014**	**2015**	**2016**	**2017**	**2018**	**2019**	**2020**
**15-44**	20.6	21.1	22.0	22.1	21.6	21.2	22.3	22.7	21.3	22.0	20.6
**45-64**	122.6	129.0	134.7	135.5	137.3	138.5	143.0	146.2	143.9	141.5	134.8
**65-75**	481.4	484.5	478.6	472.1	464.1	453.4	456.7	451.5	438.0	436.8	404.9
**>75**	1,922.0	1,912.0	1,868.2	1,816.8	1,757.8	1,715.3	1,701.1	1,649.1	1,608.0	1,602.0	1,485.4
**Total**	345.4	352.5	353.3	350.9	348.8	347.1	354.0	351.9	344.1	344.4	322.0
**Men**	**Haemorrhagic**										
	**2010**	**2011**	**2012**	**2013**	**2014**	**2015**	**2016**	**2017**	**2018**	**2019**	**2020**
**15-44**	10.9	11.3	11.2	10.7	10.8	10.6	10.8	10.0	10.0	9.6	9.6
**45-64**	60.7	60.5	61.2	60.7	59.6	58.8	59.3	59.9	56.2	55.7	54.5
**65-75**	158.8	152.8	151.4	147.0	147.2	147.1	136.6	139.0	131.1	124.4	117.7
**>75**	372.9	379.7	374.7	385.6	382.6	385.2	375.7	366.1	357.8	337.8	306.1
**Total**	74.0	75.0	75.9	77.0	77.8	78.4	77.9	78.3	75.6	73.0	68.9
**Women**	**Haemorrhagic**										
	**2010**	**2011**	**2012**	**2013**	**2014**	**2015**	**2016**	**2017**	**2018**	**2019**	**2020**
**15-44**	10.9	11.4	10.4	10.1	9.5	9.4	9.4	9.1	8.3	9.2	8.3
**45-64**	46.6	47.5	48.0	47.1	47.0	45.4	44.8	45.4	44.6	42.0	41.4
**65-75**	97.2	97.4	96.0	92.6	95.0	92.7	87.7	85.0	80.5	81.2	78.6
**>75**	269.1	263.1	262.9	269.3	266.9	277.2	261.2	252.5	248.6	235.5	221.2
**Total**	65.5	66.2	66.4	67.0	67.7	68.9	66.8	65.9	64.4	62.3	59.5

Table S6: Baseline characteristics of patients hospitalized with acute ischaemic stroke in Germany (2010-2020), by sex and age group.

	Male					Female					Total				
	15-44	45-64	65-75	> 75	all	15-44	45-64	65-75	> 75	all	15-44	45-64	65-75	> 75	all
**Number of cases – N**	39,867 (2.8)	376,293 (26.1)	384,502 (26.7)	640,133 (44.4)	1,440,795 (51.0)	33,981 (2.5)	183,345 (13.2)	251,094 (18.1)	916,220 (66.2)	1,384,640 (49.0)	73,848 (2.6)	559,638 (19.8)	635,596 (22.5)	1,556,353 (55.1)	2,825,435 (100.0)
**Median age - Yr (Q1,Q3)**	39 (34,42)	58 (53,61)	70 (67,72)	81 (78,85)	73 (63,80)	38 (32,42)	57 (52,61)	71 (68,73)	83 (79,88)	79 (71,85)					76 (66,83)
**Co-morbidities**															
**Hypertension — n (%)**	14,114 (35.4)	254,965 (67.8)	298,970 (77.8)	501,798 (78.4)	1,069,847 (74.3)	9,071 (26.7)	117,643 (64.2)	196,685 (78.3)	735,152 (80.2)	1,058,551 (76.4)	23,185 (31.4)	372,608 (66.6)	495,655 (78.0)	1,236,950 (79.5)	2,128,398 (75.3)
**Atrial fibrillation — n (%)**	1,153 (2.9)	36,539 (9.7)	87,205 (22.7)	237,567 (37.1)	362,464 (25.2)	554 (1.6)	16,458 (9.0)	62,910 (25.1)	385,050 (42.0)	464,972 (33.6)	1,707 (2.3)	52,997 (9.5)	150,115 (23.6)	622,617 (40.0)	827,436 (29.3)
**Acute myocardial infarction — n (%)**	212 (0.5)	3,797 (1.0)	5,101 (1.3)	10,010 (1.6)	19,120 (1.3)	118 (0.3)	1,521 (0.8)	2,839 (1.1)	14,232 (1.6)	18,710 (1.4)	330 (0.4)	5,318 (1.0)	7,940 (1.2)	24,242 (1.6)	37,830 (1.3)
**Cancer — n (%)**	332 (0.8)	8,186 (2.2)	13,587 (3.5)	23,835 (3.7)	45,940 (3.2)	350 (1.0)	5,677 (3.1)	8,589 (3.4)	19,894 (2.2)	34,510 (2.5)	682 (0.9)	13,863 (2.5)	22,176 (3.5)	43,729 (2.8)	80,450 (2.8)
**Cerebrovascular disease — n (%)**	2,228 (5.6)	50,394 (13.4)	61,926 (16.1)	98,140 (15.3)	212,688 (14.8)	1,861 (5.5)	20,197 (11.0)	31,077 (12.4)	100,573 (11.0)	153,708 (11.1)	4,089 (5.5)	70,591 (12.6)	93,003 (14.6)	198,713 (12.8)	366,396 (13.0)
**CHD — n (%)**	1,932 (4.8)	45,439 (12.1)	71,598 (18.6)	148,151 (23.1)	267,120 (18.5)	1,079 (3.2)	12,199 (6.7)	25,745 (10.3)	123,263 (13.5)	162,286 (11.7)	3,011 (4.1)	57,638 (10.3)	97,343 (15.3)	271,414 (17.4)	429,406 (15.2)
**Chronic heart failure — n (%)**	1,101 (2.8)	18,791 (5.0)	31,291 (8.1)	86,566 (13.5)	137,749 (9.6)	483 (1.4)	7,409 (4.0)	19,434 (7.7)	145,113 (15.8)	172,439 (12.5)	1,584 (2.1)	26,200 (4.7)	50,725 (8.0)	231,679 (14.9)	310,188 (11.0)
**RV-CHF — n (%)**	179 (0.4)	2,878 (0.8)	5,387 (1.4)	18,859 (2.9)	27,303 (1.9)	113 (0.3)	1,334 (0.7)	3,876 (1.5)	33,328 (3.6)	38,651 (2.8)	292 (0.4)	4,212 (0.8)	9,263 (1.5)	52,187 (3.4)	65,954 (2.3)
**LV-CHF — n (%)**															

NYHA I	128 (0.3)	2,283 (0.6)	3,223 (0.8)	6,779 (1.1)	12,413 (0.9)	49 (0.1)	832 (0.5)	1,861 (0.7)	10,107 (1.1)	12,849 (0.9)	177 (0.2)	3,115 (0.6)	5,084 (0.8)	16.886 (1.1)	25,262 (0.9)
NYHA II	207 (0.5)	4,545 (1.2)	7,735 (2.0)	20,342 (3.2)	32,829 (2.3)	92 (0.3)	1,715 (0.9)	4,614 (1.8)	33,743 (3.7)	40,164 (2.9)	299 (0.4)	6,260 (1.1)	12,349 (1.9)	54,085 (3.5)	72,993 (2.6)
NYHA III	264 (0.7)	4,315 (1.1)	7,266 (1.9)	21,640 (3.4)	33,485 (2.3)	96 (0.3)	1,531 (0.8)	4,221 (1.7)	34,806 (3.8)	40,654 (2.9)	360 (0.5)	5,846 (1.0)	11,487 (1.8)	56,446 (3.6)	74,139 (2.6)
NYHA IV	245 (0.6)	3,315 (0.9)	5,368 (1.4)	15,523 (2.4)	24,451 (1.7)	115 (0.3)	1,420 (0.8)	3,424 (1.4)	25,201 (2.8)	30,160 (2.2)	360 (0.5)	4,735 (0.8)	8,792 (1.4)	40,724 (2.6)	54,611 (1.9)
**Chronic kidney disease — n (%)**	947 (2.4)	18,057 (4.8)	38,969 (10.1)	119,849 (18.7)	177,822 (12.3)	617 (1.8)	8,462 (4.6)	23,825 (9.5)	170,541 (18.6)	203,445 (14.7)	1,564 (2.1)	26,519 (4.7)	62,794 (9.9)	290,390 (18.7)	381,267 (13.5)
**Diabetes mellitus — n (%)**	3,320 (8.3)	91,133 (24.2)	129,826 (33.8)	200,676 (31.3)	424,955 (29.5)	2,160 (6.4)	37,701 (20.6)	73,775 (29.4)	257,544 (28.1)	371,180 (26.8)	5,480 (7.4)	128,834 (23.0)	203,601 (32.0)	458,220 (29.4)	796,135 (28.2)
**Dyslipidaemia — n (%)**	12,303 (30.9)	168,264 (44.7)	166,848 (43.4)	240,567 (37.6)	587,982 (40.8)	7,477 (22.0)	74,898 (40.9)	106,501 (42.4)	315,982 (34.5)	504,858 (36.5)	19,780 (26.8)	243,162 (43.4)	273,349 (43.0)	556,549 (35.8)	1,092,840 (38.7)
**Obesity — n (%)**	2,686 (6.7)	24,435 (6.5)	20,710 (5.4)	20,056 (3.1)	67,887 (4.7)	2,391 (7.0)	15,405 (8.4)	17,436 (6.9)	33,938 (3.7)	69,170 (5.0)	5,077 (6.9)	39,840 (7.1)	38,146 (6.0)	53,994 (3.5)	137,057 (4.9)
**Current smoking — n (%)**	5,345 (13.4)	52,097 (13.8)	22,107 (5.7)	9,125 (1.4)	88,674 (6.2)	3,497 (10.3)	19,123 (10.4)	9,740 (3.9)	5,557 (0.6)	37,917 (2.7)	8,842 (12.0)	71,220 (12.7)	31,847 (5.0)	14,682 (0.9)	126,591 (4.5)
**Peripheral artery disease — n (%)**															
No PAD	39,744 (99.7)	365,656 (97.2)	369,130 (96.0)	617,228 (96.4)	1,391,758 (96.6)	33,909 (99.8)	180,624 (98.5)	245,599 (97.8)	896.474 (97.8)	1,356,606 (98.0)	73,653 (99.7)	546,280 (97.6)	614,729 (96.7)	1,513,702 (97.3)	2,748,364 (97.3)
PAD 1-3	93 (0.2)	8,654 (2.3)	12,515 (3.3)	17,964 (2.8)	39,226 (2.7)	50 (0.1)	2,162 (1.2)	4,411 (1.8)	14,842 (1.6)	21,465 (1.6)	143 (0.2)	10,816 (1.9)	16,926 (2.7)	32,806 (2.1)	60,691 (2.1)
CLTI	30 (0.1)	1,983 (0.5)	2,857 (0.7)	4,941 (0.8)	9,811 (0.7)	22 (0.1)	559 (0.3)	1,084 (0.4)	4,904 (0.5)	6,569 (0.5)	52 (0.1)	2,542 (0.5)	3,941 (0.6)	9,845 (0.6)	16,380 (0.6)
**Prev. Ischaemic stroke – n (%)**	1,253 (3.1)	21,760 (5.8)	30,434 (7.9)	54,693 (8.5)	108,140 (7.5)	1,018 (3.0)	9,313 (5.1)	16,311 (6.5)	64,579 (7.0)	91,221 (6.6)	2,271 (3.1)	31,073 (5.6)	46,745 (7.4)	119,272 (7.7)	199,361 (7.1)

**Prev. Intracranial bleeding – n (%)**	152 (0.4)	1,992 (0.5)	2,320 (0.6)	3,332 (0.5)	7,796 (0.5)	140 (0.4)	1,217 (0.7)	1,467 (0.6)	3,672 (0.4)	6,496 (0.5)	292 (0.4)	3,209 (0.6)	3,787 (0.6)	7,004 (0.5)	14,292 (0.5)
**Ischaemic heart disease – n (%)**	2,039 (5.1)	46,893 (12.5)	73,489 (19.1)	152,249 (23.8)	274,670 (19.1)	1,142 (3.4)	12,935 (7.1)	27,100 (10.8)	130,987 (14.3)	172,164 (12.4)	3,181 (4.3)	59,828 (10.7)	100,589 (15.8)	283,236 (18.2)	446,834 (15.8)
**Dementia – n (%)**	13 (0.03)	2,000 (0.5)	10,110 (2.6)	48,587 (7.6)	60,710 (4.2)	10 (0.03)	877 (0.5)	6,537 (2.6)	88,197 (9.6)	95,621 (6.9)	23 (0.03)	2,877 (0.5)	16,647 (2.6)	136,784 (8.8)	156,331 (5.5)
**Prev. CABG – n (%)**	85 (0.2)	5,490 (1.5)	12,588 (3.3)	27,151 (4.2)	45,314 (3.1)	29 (0.1)	875 (0.5)	2,848 (1.1)	11,664 (1.3)	15,416 (1.1)	114 (0.2)	6,365 (1.1)	15,436 (2.4)	38,815 (2.5)	60,730 (2.1)
**Prev. Valve replacement– n (%)**	367 (0.9)	2,472 (0.7)	2,966 (0.8)	5,708 (0.9)	11,513 (0.8)	155 (0.5)	888 (0.5)	1,440 (0.6)	5,396 (0.6)	7,879 (0.6)	522 (0.7)	3,360 (0.6)	4,406 (0.7)	11,104 (0.7)	19,392 (0.7)

CABG indicates coronary artery bypass graft; CHD, coronary heart disease; CHF, congestive heart failure; CLTI, chronic limb-threatening ischaemia; LV, left ventricular; NYHA, New York Heart Association; PAD, peripheral artery disease; Prev., previous; Q1, 25% quartile; Q3, 75% quartile; RV, right ventricular.

Table S7: Baseline characteristics of all patients hospitalized with acute haemorrhagic stroke in Germany (2010-2020) according to sex.

	Male sex	Female sex	Total	P value
**Number of cases – N**	288,159	261,563	549,722	***
**Median age - Yr (Q1,Q3)**	72 (60,80)	75 (62,83)	74 (61,81)	<0.001
**Co-morbidities**				
**Hypertension — n (%)**	191,307 (66.4)	167,967 (64.2)	359,274 (65.4)	<0.001
**Atrial fibrillation — n (%)**	59,039 (20.5)	47,762 (18.3)	106,801 (19.4)	<0.001
**Acute myocardial infarction — n (%)**	2,429 (0.8)	1,934 (0.7)	4,363 (0.8)	<0.001
**Cancer — n (%)**	11,244 (3.9)	7,387 (2.8)	18,631 (3.4)	<0.001
**Cerebrovascular disease — n (%)**	7,650 (2.7)	5,504 (2.1)	13,154 (2.4)	<0.001
**CHD — n (%)**	36,905 (12.8)	16,904 (6.5)	53,809 (9.8)	<0.001
**Chronic heart failure — n (%)**	19,704 (6.8)	17,085 (6.5)	36,789 (6.7)	<0.001
**RV-CHF — n (%)**	4,232 (1.5)	3,730 (1.4)	7,962 (1.4)	0.187
**LV-CHF — n (%)**				<0.001
**NYHA I**	1,173 (0.4)	991 (0.4)	2,164 (0.4)	
**NYHA II**	4,001 (1.4)	3,636 (1.4)	7,637 (1.4)	
**NYHA III**	4,703 (1.6)	3,865 (1.5)	8,568 (1.6)	
**NYHA IV**	4,175 (1.4)	3,171 (1.2)	7,346 (1.3)	
**Chronic kidney disease — n (%)**	27,257 (9.5)	20,712 (7.9)	47,969 (8.7)	<0.001
**Diabetes mellitus — n (%)**	55,083 (19.1)	37,891 (14.5)	92,974 (16.9)	<0.001
**Dyslipidemia — n (%)**	38,654 (13.4)	28,533 (10.9)	67,187 (12.2)	<0.001
**Obesity — n (%)**	10,372 (3.6)	9,395 (3.6)	19,767 (3.6)	0.881
**Current smoking — n (%)**	6,966 (2.4)	3,952 (1.5)	10,918 (2.0)	<0.001
**Peripheral artery disease – n (%)**				<0.001
**PAD 1-3**	3,563 (1.2)	1,631 (0.6)	5,194 (0.9)	
**CLTI**	1,042 (0.4)	503 (0.2)	1,545 (0.3)	
**Prev. Intracranial bleeding – n (%)**	5,560 (1.9)	5,196 (2.0)	10,756 (2.0)	0.127
**Ischaemic heart disease – n (%)**	38,177 (13.2)	18,113 (6.9)	56,290 (10.2)	<0.001
**Dementia – n (%)**	8,304 (2.9)	11,022 (4.2)	19,326 (3.5)	<0.001
**Prev. CABG – n (%)**	6,571 (2.3)	1,520 (0.6)	8,091 (1.5)	<0.001
**Prev. Valve replacement– n (%)**	2,697 (0.9)	1,373 (0.5)	4,070 (0.7)	<0.001

CABG indicates coronary artery bypass graft; CHD, coronary heart disease; CHF, congestive heart failure; CTLI, chronic limb-threatening ischaemia; LV, left ventricular; NYHA, New York Heart Association; PAD, peripheral artery disease; Prev., previous; Q1, 25% quartile; Q3, 75% quartile; RV, right ventricular.

Table S8: Baseline characteristics of patients hospitalized with acute haemorrhagic stroke in Germany (2010-2020), by sex and by age group.

	Male					Female					Total				
	15-44	45-64	65-75	> 75	all	15-44	45-64	65-75	> 75	all	15-44	45-64	65-75	> 75	all
**Number of cases – N**	17,290 (6.0)	78,563 (27.3)	68,991 (23.9)	123,315 (42.8)	288,159 (100.0)	15,181 (5.8)	60,742 (23.2)	49,233 (18.8)	136,407 (52.2)	261,563 (100.0)	32,471 (5.9)	139,305 (25.3)	118,224 (21.5)	259,722 (47.2)	549,722 (100.0)
**Median age - Yr (Q1,Q3)**	38 (31,42)	56 (52,61)	70 (68,73)	81 (78,85)	72 (60,80)	38 (31,42)	56 (51,60)	70 (68,73)	82 (79,86)	75 (62,83)					74 (61,81)
**Co-morbidities**															
**Hypertension — n (%)**	5,819 (33.7)	49,038 (62.4)	48730 (70.6)	87,720 (71.1)	191,307 (66.4)	3,726 (24.5)	31,955 (52.6)	33,251 (67.5)	99,035 (72.6)	167,967 (64.2)	9,545 (29.4)	80,993 (58.1)	81,981 (69.3)	186,755 (71.9)	359,274 (65.4)
**Atrial fibrillation — n (%)**	211 (1.2)	5,147 (6.6)	14,335 (20.8)	39,346 (31.9)	59,039 (20.5)	95 (0.6)	2,328 (3.8)	8,050 (16.4)	37,289 (27.3)	47,762 (18.3)	306 (0.9)	7,475 (5.4)	22,385 (18.9)	76,635 (29.5)	106,801 (19.4)
**Acute myocardial infarction — n (%)**	85 (0.5)	634 (0.8)	588 (0.9)	1,122 (0.9)	2,429 (0.8)	45 (0.3)	400 (0.7)	384 (0.8)	1,105 (0.8)	1,934 (0.7)	130 (0.4)	1,034 (0.7)	972 (0.8)	2,227 (0.9)	4,363 (0.8)
**Cancer — n (%)**	352 (2.0)	2,379 (3.0)	3,268 (4.7)	5,245 (4.3)	11,244 (3.9)	258 (1.7)	1,759 (2.9)	2,041 (4.1)	3,329 (2.4)	7,387 (2.8)	610 (1.9)	4,138 (3.0)	5,309 (4.5)	8,574 (3.3)	18,631 (3.4)
**Cerebrovascular disease — n (%)**	104 (0.6)	1,491 (1.9)	2,163 (3.1)	3,892 (3.2)	7,650 (2.7)	131 (0.9)	907 (1.5)	1,078 (2.2)	3,388 (2.5)	5,504 (2.1)	235 (0.7)	2,398 (1.7)	3,241 (2.7)	7,280 (2.8)	13,154 (2.4)
**CHD — n (%)**	181 (1.0)	4,977 (6.3)	9,477 (13.7)	22,270 (18.1)	36,905 (12.8)	87 (0.6)	1,514 (2.5)	2,946 (6.0)	12,357 (9.1)	16,904 (6.5)	268 (0.8)	6,491 (4.7)	12,423 (10.5)	34,627 (13.3)	53,809 (9.8)
**Chronic heart failure — n (%)**	330 (1.9)	2,918 (3.7)	4,336 (6.3)	12,120 (9.8)	19,704 (6.8)	193 (1.3)	1,498 (2.5)	2,612 (5.3)	12,782 (9.4)	17,085 (6.5)	523 (1.6)	4,416 (3.2)	6,948 (5.9)	24,902 (9.6)	36,789 (6.7)
**RV-CHF — n (%)**	49 (0.3)	582 (0.7)	874 (1.3)	2,727 (2.2)	4,232 (1.5)	31 (0.2)	283 (0.5)	574 (1.2)	2,842 (2.1)	3,730 (1.4)	80 (0.2)	865 (0.6)	1,448 (1.2)	5,569 (2.1)	7,962 (1.4)
**LV-CHF — n (%)**															
**NYHA I**	24 (0.1)	179 (0.2)	278 (0.4)	692 (0.6)	1,173 (0.4)	12 (0.1)	76 (0.1)	158 (0.3)	745 (0.5)	991 (0.4)	36 (0.1)	255 (0.2)	436 (0.4)	1,437 (0.6)	2,164 (0.4)

**NYHA II**	44 (0.3)	516 (0.7)	844 (1.2)	2,597 (2.1)	4,001 (1.4)	21 (0.1)	211 (0.3)	503 (1.0)	2,901 (2.1)	3,636 (1.4)	65 (0.2)	727 (0.5)	1,347 (1.1)	5,498 (2.1)	7,637 (1.4)
**NYHA III**	55 (0.3)	630 (0.8)	1,020 (1.5)	2,998 (2.4)	4.703 (1.6)	35 (0.2)	276 (0.5)	602 (1.2)	2,952 (2.2)	3,865 (1.5)	90 (0.3)	906 (0.7)	1,622 (1.4)	5,950 (2.3)	8,568 (1.6)
**NYHA IV**	109 (0.6)	732 (0.9)	988 (1.4)	2,346 (1.9)	4.175 (1.4)	63 (0.4)	408 (0.7)	494 (1.0)	2,206 (1.6)	3,171 (1.2)	172 (0.5)	1,140 (0.8)	1,482 (1,3)	4,552 (1.8)	7,346 (1.3)
**Chronic kidney disease — n (%)**	593 (3.4)	4,048 (5.2)	5,816 (8.4)	16,800 (13.6)	27,257 (9.5)	257 (1.7)	1,856 (3.1)	3,009 (6.1)	15,590 (11.4)	20,712 (7.9)	850 (2.6)	5,904 (4.2)	8,825 (7.5)	32,390 (12.5)	47,969 (8.7)
**Diabetes mellitus — n (%)**	653 (3.8)	11,031 (14.0)	16,174 (23.4)	27,225 (22.1)	55,083 (19.1)	392 (2.6)	5,073 (8.4)	7,930 (16.1)	24,496 (18.0)	37,891 (14.5)	1,045 (3.2)	16,104 (11.6)	24,104 (20.4)	51,721 (19.9)	92,974 (16.9)
**Dyslipidemia — n (%)**	613 (3.5)	7,705 (9.8)	10,644 (15.4)	19,692 (16.0)	38,654 (13.4)	411 (2.7)	4,287 (7.1)	5,992 (12.2)	17,843 (13.1)	28,533 (10.9)	1,024 (3.2)	11,992 (8.6)	16,636 (14.1)	37,535 (14.5)	67,187 (12.2)
**Obesity — n (%)**	834 (4.8)	4,164 (5.3)	2,804 (4.1)	2,570 (2.1)	10,372 (3.6)	590 (3.9)	3,038 (5.0)	2,357 (4.8)	3,410 (2.5)	9,395 (3.6)	1,424 (4.4)	7,202 (5.2)	5,161 (4.4)	5,980 (2.3)	19,767 (3.6)
**Current smoking — n (%)**	804 (4.7)	3,915 (5.0)	1,466 (2.1)	781 (0.6)	6,966 (2.4)	499 (3.3)	2,275 (3.7)	762 (1.5)	416 (0.3)	3,952 (1.5)	1,303 (4.0)	6,190 (4.4)	2,228 (1.9)	1,197 (0.5)	10,918 (2.0)
**Peripheral artery disease — n (%)**															
**No PAD**	17,278 (99.93)	77,756 (99.0)	67,650 (98.1)	120,870 (98.0)	283,554 (98.4)	15,173 (99.95)	60,487 (99.6)	48,723 (99.0)	135,046 (99.0)	259,429 (99.2)	32,451 (99.94)	138,243 (99.2)	116,373 (98.4)	255,916 (98.5)	542,983 (98.8)
**PAD 1-3**	9 (0.05)	607 (0.8)	1,067 (1.6)	1,880 (1.5)	3,563 (1.2)	4 (0.03)	204 (0.3)	384 (0.8)	1,039 (0.8)	1,631 (0.6)	13 (0.04)	811 (0.6)	1,451 (1.2)	2,919 (1.1)	5,194 (0.9)
**CLTI**	3 (0.02)	200 (0.3)	274 (0.4)	565 (0.5)	1,042 (0.4)	4 (0.03)	51 (0.1)	126 (0.3)	322 (0.2)	503 (0.2)	7 (0.02)	251 (0.2)	400 (0.3)	887 (0.3)	1,545 (0.3)
**Prev. Ischaemic stroke – n (%)**	0 (0.0)	0 (0.0)	0 (0.0)	0 (0.0)	0 (0.0)	0 (0.0)	0 (0.0)	0 (0.0)	0 (0.0)	0 (0.0)	0 (0.0)	0 (0.0)	0 (0.0)	0 (0.0)	0 (0.0)
**Prev. Intracranial bleeding – n (%)**	340 (2.0)	1,619 (2.1)	1,542 (2.2)	2,059 (1.7)	5,560 (1.9)	316 (2.1)	1,356 (2.2)	1,161 (2.4)	2,363 (1.7)	5,196 (2.0)	656 (2.0)	2,975 (2.1)	2,703 (2.3)	4,422 (1.7)	10,756 (2.0)
**Ischaemic heart disease – n (%)**	238 (1.4)	5,356 (6.8)	9,755 (14.1)	22,828 (18.5)	38,177 (13.2)	121 (0.8)	1,784 (2.9)	3,168 (6.4)	13,040 (9.6)	18,113 (6.9)	359 (1.1)	7,140 (5.1)	12,923 (10.9)	35,868 (13.8)	56,290 (10.2)

**Dementia – n (%)**	-	213 (0.3)	1,237 (1.8)	6,852 (5.6)	8,304 (2.9)	-	129 (0.2)	1,003 (2.0)	9,889 (7.2)	11,022 (4.2)	-	342 (0.2)	2,240 (1.9)	16,741 (6.4)	19,326 (3.5)
**Prev. CABG – n (%)**	-	559 (0.7)	1,699 (2.5)	4,305 (3.5)	6,571 (2.3)	-	107 (0.2)	284 (0.6)	1,127 (0.8)	1,520 (0.6)	10 (0.03)	666 (0.5)	1,983 (1.7)	5,432 (2.1)	8,091 (1.5)
**Prev. Valve replacement– n (%)**	62 (0.4)	539 (0.7)	755 (1.1)	1,341 (1.1)	2,697 (0.9)	33 (0.2)	202 (0.3)	308 (0.6)	830 (0.6)	1,373 (0.5)	95 (0.3)	741 (0.5)	1,063 (0.9)	2,171 (0.8)	4,070 (0.7)

CABG indicates coronary artery bypass graft; CHD, coronary heart disease; CHF, congestive heart failure; CLTI, chronic limb threatening ischaemia; LV, left ventricular; NYHA, New York Heart Association; PAD, peripheral artery disease; prev., previous; Q1, 25% quartile; Q3, 75% quartile; RV, right ventricular
A dash - indicates no data due to data protection reasons.

Table S9. Acute therapies and complications experienced by patients hospitalized with acute ischaemic stroke in Germany (2010-2020) according to sex and age group.

	Male					Female					Total				
	15-44	45-64	65-75	> 75	all	15-44	45-64	65-75	> 75	all	15-44	45-64	65-75	> 75	all
**Carotid EA – n (%)**	121 (0.3)	7,674 (2.0)	10,499 (2.7)	13,597 (2.1)	31,891 (2.2)	134 (0.4)	3,359 (1.8)	4,207 (1.7)	6,965 (0.8)	14,665 (1.1)	255 (0.3)	11,033 (2.0)	14,706 (2.3)	20,562 (1.3)	46,556 (1.6)
**Carotid stent – n (%)**	266 (0.7)	6,226 (1.7)	5,594 (1.5)	5,847 (0.9)	17,933 (1.2)	235 (0.7)	2,512 (1.4)	2,201 (0.9)	3,040 (0.3)	7,988 (0.6)	501 (0.7)	8,738 (1.6)	7,795 (1.2)	8,887 (0.6)	25,921 (0.9)
**Carotid interponate – n (%)**	-	78 (0.02)	120 (0.03)	127 (0.02)	325 (0.02)	-	27 (0.01)	27 (0.01)	68 (0.01)	123 (0.01)	-	105 (0.02)	147 (0.02)	195 (0.01)	448 (0.02)
**Craniectomy – n (%)**	652 (1.6)	3,123 (0.8)	1,148 (0.3)	510 (0.1)	5,433 (0.4)	681 (2.0)	2,227 (1.2)	971 (0.4)	562 (0.1)	4,441 (0.3)	1,333 (1.8)	5,350 (1.0)	2,119 (0.3)	1,072 (0.1)	9,874 (0.3)
**Evacuation of extracranial haemorrhage – n (%)**	93 (0.2)	550 (0.1)	594 (0.2)	1,303 (0.2)	2,540 (0.2)	95 (0.3)	443 (0.2)	367 (0.1)	706 (0.1)	1,611 (0.1)	188 (0.3)	993 (0.2)	961 (0.2)	2,009 (0.1)	4,151 (0.1)
**Evacuation of intracranial haemorrhage – n (%)**	0 (0.0)	12 (0.00)	7 (0.00)	11 (0.00)	30 (0.00)	3 (0.00)	8 (0.00)	8 (0.00)	5 (0.00)	24 (0.00)	3 (0.00)	20 (0.00)	15 (0.00)	16 (0.00)	54 (0.00)
**Thrombectomy n (%)**															
**Intracranial only**	1,589 (4.0)	13,020 (3.5)	10,942 (2.8)	17,765 (2.8)	43,316 (3.0)	1,732 (5.1)	7,153 (3.9)	9,422 (3.8)	30,735 (3.4)	49,042 (3.5)	3,321 (4.5)	20,173 (3.6)	20,364 (3.2)	48,500 (3.1)	92,358 (3.3)

**Extracranial only**	13 (0.03)	218 (0.06)	171 (0.04)	179 (0.03)	581 (0.04)	22 (0.06)	86 (0.05)	94 (0.04)	184 (0.02)	386 (0.03)	35 (0.05)	304 (0.05)	265 (0.04)	363 (0.02)	967 (0.03)
**both**	49 (0.1)	463 (0.1)	316 (0.1)	374 (0.1)	1,202 (0.1)	45 (0.1)	211 (0.1)	179 (0.1)	488 (0.1)	923 (0.1)	94 (0.1)	674 (0.1)	495 (0.1)	862 (0.1)	2,125 (0.1)
**Thrombectomy intracranial – n (%)**	1,638 (4.1)	13,483 (3.6)	11,258 (2.9)	18,139 (2.8)	44,518 (3.1)	1,777 (5.2)	7,364 (4.0)	9,601 (3.8)	31,223 (3.4)	49,965 (3.6)	3,415 (4.6)	20,847 (3.7)	20,859 (3.3)	49,362 (3.2)	94,483 (3.3)
**Thrombectomy extracranial – n (%)**	62 (0.2)	681 (0.2)	487 (0.1)	553 (0.1)	1,783 (0.1)	67 (0.2)	297 (0.2)	273 (0.1)	672 (0.1)	1,309 (0.1)	129 (0.2)	978 (0.2)	760 (0.1)	1,225 (0.1)	3,092 (0.1)
**PCI – n (%)**	187 (0.5)	1,834 (0.5)	1,685 (0.4)	2,095 (0.3)	5,801 (0.4)	133 (0.4)	556 (0.3)	637 (0.3)	1,588 (0.2)	2,914 (0.2)	320 (0.4)	2,390 (0.4)	2,322 (0.4)	3,683 (0.2)	8,715 (0.3)
**CABG – n (%)**	-	53 (0.01)	69 (0.02)	43 (0.01)	166 (0.01)	-	10 (0.01)	23 (0.01)	15 (0.00)	52 (0.00)	5 (0.01)	63 (0.01)	92 (0.01)	58 (0.00)	218 (0.01)
**Renal replacement therapy – n (%)**	225 (0.6)	2,020 (0.5)	2,603 (0.7)	3,975 (0.6)	8,823 (0.6)	153 (0.5)	951 (0.5)	1,464 (0.6)	3,178 (0.3)	5,746 (0.4)	378 (0.5)	2,971 (0.5)	4,067 (0.6)	7,153 (0.5)	14,569 (0.5)
**Systemic Thrombolysis – n (%)**	5,979 (15.0)	48,662 (12.9)	45,215 (11.8)	72,765 (11.4)	172,621 (12.0)	4,946 (14.6)	22,456 (12.2)	29,857 (11.9)	101,119 (11.0)	158,378 (11.4)	10,925 (14.8)	71,118 (12.7)	75,072 (11.8)	173,884 (11.2)	330,999 (11.7)
**Selective Thrombolysis – n (%)**	340 (0.9)	2,016 (0.5)	1,707 (0.4)	1,967 (0.3)	6,030 (0.4)	420 (1.2)	1,276 (0.7)	1,343 (0.5)	2,849 (0.3)	5,888 (0.4)	760 (1.0)	3,292 (0.6)	3,050 (0.5)	4,816 (0.3)	11,918 (0.4)
**Intracerebral Thrombolysis – n (%)**	14 (0.04)	101 (0.03)	62 (0.02)	66 (0.01)	243 (0.02)	20 (0.06)	72 (0.04)	43 (0.02)	60 (0.01)	195 (0.01)	34 (0.05)	173 (0.03)	105 (0.02)	126 (0.01)	438 (0.02)
**GpIIb/IIIa – n (%)**	125 (0.3)	1,044 (0.3)	636 (0.2)	680 (0.1)	2,485 (0.2)	130 (0.4)	521 (0.3)	386 (0.2)	535 (0.1)	1,572 (0.1)	255 (0.3)	1,565 (0.3)	1,022 (0.2)	1,215 (0.1)	4,057 (0.1)

**Haemorrhagic stroke (as secondary diagnosis) – n (%)**	1,208 (3.0)	10,326 (2.7)	10,667 (2.8)	19,070 (3.0)	41,271 (2.9)	1,395 (4.1)	6,987 (3.8)	7,870 (3.1)	24,534 (2.7)	40,786 (2.9)	2,603 (3.5)	17,313 (3.1)	18,537 (2.9)	43,604 (2.8)	82,057 (2.9)
**Bleeding – n (%)**	453 (1.1)	4,641 (1.2)	5,480 (1.4)	10,497 (1.6)	21,071 (1.5)	423 (1.2)	2,457 (1.3)	3,437 (1.4)	12,322 (1.3)	18,639 (1.3)	876 (1.2)	7,098 (1.3)	8,917 (1.4)	22,819 (1.5)	39,710 (1.4)
**Blood transfusion– n (%)**	1,007 (2.5)	7,104 (1.9)	7,638 (2.0)	12,612 (2.0)	28,361 (2.0)	1,521 (4.5)	6,607 (3.6)	6,247 (2.5)	18,548 (2.0)	32,923 (2.4)	2,528 (3.4)	13,711 (2.4)	13,885 (2.2)	31,160 (2.0)	61,284 (2.2)
**Blood transfusion or bleeding event – n (%)**	1,300 (3.3)	10,524 (2.8)	11,828 (3.1)	20,925 (3.3)	44,577 (3.1)	1,760 (5.2)	8,161 (4.5)	8,779 (3.5)	27,920 (3.0)	46,620 (3.4)	3,060 (4.1)	18,685 (3.3)	20,607 (3.2)	48,845 (3.1)	91,197 (3.2)
**AKI – n (%)**	431 (1.1)	4,697 (1.2)	6,674 (1.7)	16,242 (2.5)	28,044 (1.9)	217 (0.6)	2,002 (1.1)	3,656 (1.5)	17,878 (2.0)	23,753 (1.7)	648 (0.9)	6,699 (1.2)	10,330 (1.6)	34,120 (2.2)	51,797 (1.8)
**Sepsis – n (%)**	562 (1.4)	4,935 (1.3)	5,639 (1.5)	9,225 (1.4)	20,361 (1.4)	410 (1.2)	2,230 (1.2)	2,596 (1.0)	8,036 (0.9)	13,272 (1.0)	972 (1.3)	7,165 (1.3)	8,235 (1.3)	17,261 (1.1)	33,633 (1.2)
**AKI or need for RRT – n (%)**	551 (1.4)	5,894 (1.6)	8,418 (2.2)	19,191 (3.0)	34,054 (2.4)	302 (0.9)	2,630 (1.4)	4,721 (1.9)	20,335 (2.2)	27,988 (2.0)	853 (1.2)	8,524 (1.5)	13,139 (2.1)	39,526 (2.5)	62,042 (2.2)
**Cardiac resuscitation – n (%)**	138 (0.3)	1,817 (0.5)	2,317 (0.6)	3,941 (0.6)	8,213 (0.6)	97 (0.3)	903 (0.5)	1,295 (0.5)	3,660 (0.4)	5,955 (0.4)	235 (0.3)	2,720 (0.5)	3,612 (0.6)	7,601 (0.5)	14,168 (0.5)
**Mechanical ventilation – n (%)**	3,118 (7.8)	23,104 (6.1)	20,952 (5.4)	27,249 (4.3)	74,423 (5.2)	2,954 (8.7)	13,948 (7.6)	13,845 (5.5)	30,894 (3.4)	61,641 (4.5)	6,072 (8.2)	37,052 (6.6)	34,797 (5.5)	58,143 (3.7)	136,064 (4.8)
**Median duration of**	193 (361)	166 (355)	132.5 (328)	71 (214)	116 (305)	193 (379)	188 (362)	105 (281)	45 (180)	75 (244)					96 (279)

**ventilation – h (IQR)**															
**In Hospital Death – n (%)**	623 (1.6)	9,021 (2.4)	16,208 (4.2)	55,646 (8.7)	81,498 (5.7)	639 (1.9)	5,666 (3.1)	11,754 (4.7)	97,521 (10.6)	115,580 (8.3)	1,262 (1.7)	14,687 (2.6)	27,962 (4.4)	153,167 (9.8)	197,078 (7.0)
**Mean length of hospital stay – days (SD)**	10.8 (16.2)	11.4 (15.2)	12.2 (14.8)	12.4 (12.5)	12.0 (14.0)	11.2 (16.6)	11.9 (15.8)	12.4 (13.9)	12.3 (11.0)	12.2 (12.4)					12.1 (13.3)
**Mean charges per case – EUR (SD)**	7,551 (13,433)	7,106 (11,878)	6,784 (10,432)	6,243 (7,537)	6,649 (9,811)	7,999 (14,743)	7,664 (13,219)	6,613 (9,343)	5,784 (5,494)	6,235 (8,035)					6,446 (8,986)

AKI indicates acute kidney injury; CABG, coronary artery bypass grafting; EA, endarterectomy; IQR, interquartile range; PCI, percutaneous coronary intervention; RRT, renal replacement therapy; SD, standard deviation
A dash - indicates no data due to data protection reasons.

Table S10: Acute therapies and complications experienced by patients hospitalized with acute haemorrhagic stroke in Germany (2010-2020) according to sex.

	Male	Female	Total	P value
**Craniectomy – n (%)**	5,442 (1.9)	4,994 (1.9)	10,436 (1.9)	0.573
**Evacuation of extracranial haemorrhage – n (%)**	35,487 (12.3)	16,857 (6.4)	52,344 (9.5)	<0.001
**ICH evacuation – n (%)**	190 (0.07)	128 (0.05)	318 (0.06)	0.009
**PCI – n (%)**	263 (0.1)	158 (0.1)	421 (0.1)	<0.001
**CABG – n (%)**	9 (0.00)	6 (0.00)	15 (0.00)	0.557
**Renal replacement therapy – n (%)**	3,188 (1.1)	1,637 (0.6)	4,825 (0.9)	<0.001
**Bleeding – n (%)**	6,072 (2.1)	4,987 (1.9)	11,059 (2.0)	<0.001
**Blood transfusion– n (%)**	14,794 (5.1)	16,349 (6.3)	31,143 (5.7)	<0.001
**Blood transfusion or bleeding event – n (%)**	18,997 (6.6)	19,545 (7.5)	38,542 (7.0)	<0.001
**AKI – n (%)**	8,833 (3.1)	4,734 (1.8)	13,567 (2.5)	<0.001
**Sepsis – n (%)**	8,909 (3.1)	4,892 (1.9)	13,801 (2.5)	<0.001
**AKI or need for RRT – n (%)**	10,437 (3.6)	5,609 (2.1)	16,046 (2.9)	<0.001
**Cardiac arrest – n (%)**	3,257 (1.1)	2,563 (1.0)	5,820 (1.1)	<0.001
**Mechanical ventilation – n (%)**	64,026 (22.2)	59,302 (22.7)	123,328 (22.4)	<0.001
**Median duration of ventilation – h (IQR)**	97 (315)	66 (262)	79 (290)	<0.001
**In Hospital Death – n (%)**	45,818 (15.9)	52,037 (19.9)	97,855 (17.8)	<0.001
**Mean length of hospital stay – days (SD)**	15.6 (21.0)	14.9 (19.2)	15.3 (20.2)	<0.001
**Mean charges per case – EUR (SD)**	10,908 (18,869)	10,090 (16,691)	10,518 (17,868)	<0.001

AKI indicates acute kidney injury; BMS, bare metal stent; CABG OP, coronary artery bypass graft off pump; DES, drug-eluting stent; EA, endarterectomy; ICH, intracerebral haemorrhage; IQR, interquartile range; PCI, percutaneous coronary intervention; RRT, renal replacement therapy; SD, standard deviation.

Table S11: Acute therapies and complications experienced by patients hospitalized with acute haemorrhagic stroke in Germany (2010-2020) according to sex and age group.

	Male					Female					Total				
	15-44	45-64	65-75	> 75	all	15-44	45-64	65-75	> 75	all	15-44	45-64	65-75	> 75	all
**Carotid EA – n (%)**	0 (0.0)	12 (0.02)	12 (0.02)	14 (0.01)	38 (0.01)	0 (0.0)	7 (0.01)	9 (0.02)	6 (0.00)	22 (0.01)	0 (0.0)	19 (0.01)	21 (0.02)	20 (0.01)	60 (0.01)
**Carotid stent – n (%)**	10 (0.06)	26 (0.03)	18 (0.03)	10 (0.01)	64 (0.02)	17 (0.11)	43 (0.07)	8 (0.02)	17 (0.01)	85 (0.03)	27 (0.08)	69 (0.05)	26 (0.02)	27 (0.01)	149 (0.03)
**Carotid interposition graft – n (%)**	0 (0.0)	-	-	0 (0.0)	0 (0.0)	0 (0.0)	-	-	0 (0.0)	3 (0.0)	0 (0.0)	-	-	0 (0.0)	3 (0.0)
**Craniectomy – n (%)**	591 (3.4)	1,912 (2.4)	1,319 (1.9)	1,620 (1.3)	5,442 (1.9)	590 (3.9)	1,943 (3.2)	1,144 (2.3)	1,317 (1.0)	4,994 (1.9)	1,181 (3.6)	3,855 (2.8)	2,463 (2.1)	2,937 (1.1)	10,436 (1.9)
**Evacuation of extracranial haemorrhage – n (%)**	907 (5.2)	5,981 (7.6)	9,161 (13.3)	19,438 (15.8)	35,487 (12.3)	522 (3.4)	2,636 (4.3)	3,788 (7.7)	9,911 (7.3)	16,857 (6.4)	1,429 (4.4)	8,617 (6.2)	12,949 (11.0)	29,349 (11.3)	52,344 (9.5)
**Evacuation of intracranial haemorrhage – n (%)**	21 (0.12)	66 (0.08)	49 (0.07)	54 (0.04)	190 (0.07)	3 (0.02)	46 (0.08)	36 (0.07)	43 (0.03)	128 (0.05)	24 (0.07)	112 (0.08)	85 (0.07)	97 (0.04)	318 (0.06)
**PCI – n (%)**	3 (0.02)	68 (0.1)	79 (0.1)	113 (0.1)	263 (0.1)	5 (0.03)	35 (0.1)	43 (0.1)	75 (0.1)	158 (0.1)	8 (0.02)	103 (0.1)	122 (0.1)	188 (0.1)	421 (0.1)

**CABG – n (%)**	0 (0.0)	-	-	-	9 (0.00)	0 (0.0)	-	-	-	6 (0.00)	0 (0.0)	4 (0.00)	6 (0.01)	5 (0.00)	15 (0.00)
**Renal replacement therapy – n (%)**	256 (1.5)	1,226 (1.6)	817 (1.2)	889 (0.7)	3,188 (1.1)	132 (0.9)	593 (1.0)	393 (0.8)	519 (0.4)	1,637 (0.6)	388 (1.2)	1,819 (1.3)	1,210 (1.0)	1,408 (0.5)	4,825 (0.9)
**Systemic Thrombolysis – n (%)**	44 (0.3)	227 (0.3)	128 (0.2)	161 (0.1)	560 (0.2)	53 (0.3)	214 (0.4)	109 (0.2)	193 (0.1)	569 (0.2)	97 (0.3)	441 (0.3)	237 (0.2)	354 (0.1)	1,129 (0.2)
**Selective Thrombolysis – n (%)**	68 (0.4)	213 (0.3)	62 (0.1)	49 (0.04)	392 (0.1)	101 (0.7)	371 (0.6)	77 (0.2)	80 (0.06)	629 (0.2)	169 (0.5)	584 (0.4)	139 (0.1)	129 (0.05)	1,021 (0.2)
**Intracerebral Thrombolysis – n (%)**	69 (0.4)	419 (0.5)	305 (0.4)	353 (0.3)	1,146 (0.4)	44 (0.3)	304 (0.5)	228 (0.5)	357 (0.3)	933 (0.4)	113 (0.3)	723 (0.5)	533 (0.5)	710 (0.3)	2,079 (0.4)
**GpIIb/IIIa – n (%)**	57 (0.3)	171 (0.2)	43 (0.1)	28 (0.02)	299 (0.1)	105 (0.7)	335 (0.6)	100 (0.2)	55 (0.04)	595 (0.2)	162 (0.5)	506 (0.4)	143 (0.1)	83 (0.03)	894 (0.2)
**Haemorhagic stroke (as secondary diagnosis) – n (%)**	2,203 (12.7)	9,801 (12.5)	7,761 (11.2)	13,010 (10.6)	32,775 (11.4)	2,281 (15.0)	9,589 (15.8)	6,666 (13.5)	14,730 (10.8)	33,266 (12.7)	4,484 (13.8)	19,390 (13.9)	14,427 (12.2)	27,740 (10.7)	66,041 (12.0)
**Bleeding – n (%)**	450 (2.6)	1,824 (2.3)	1,507 (2.2)	2,291 (1.9)	6,072 (2.1)	402 (2.6)	1,519 (2.5)	1,101 (2.2)	1,965 (1.4)	4,987 (1.9)	852 (2.6)	3,343 (2.4)	2,608 (2.2)	4,256 (1.6)	11,059 (2.0)
**Blood transfusion– n (%)**	1,086 (6.3)	4,807 (6.1)	3,831 (5.6)	5,070 (4.1)	14,794 (5.1)	1,500 (9.9)	5,646 (9.3)	3,739 (7.6)	5,464 (4.0)	16,349 (6.3)	2,586 (8.0)	10,453 (7.5)	7,570 (6.4)	10,534 (4.1)	31,143 (5.7)

**Blood transfusion or bleeding event – n (%)**	1,369 (7.9)	5,977 (7.6)	4,889 (7.1)	6,762 (5.5)	18,997 (6.6)	1,724 (11.4)	6,524 (10.7)	4,405 (8.9)	6,892 (5.1)	19,545 (7.5)	3,093 (9.5)	12,501 (9.0)	9,294 (7.9)	13,654 (5.3)	38,542 (7.0)
**AKI – n (%)**	456 (2.6)	2,643 (3.4)	2,076 (3.0)	3,658 (3.0)	8,833 (3.1)	232 (1.5)	1,044 (1.7)	998 (2.0)	2,460 (1.8)	4,734 (1.8)	688 (2.1)	3,687 (2.6)	3,074 (2.6)	6,118 (2.4)	13,567 (2.5)
**Sepsis – n (%)**	628 (3.6)	3,062 (3.9)	2,313 (3.4)	2,906 (2.4)	8,909 (3.1)	384 (2.5)	1,715 (2.8)	1,076 (2.2)	1,717 (1.3)	4,892 (1.9)	1,012 (3.1)	4,777 (3.4)	3,389 (2.9)	4,623 (1.8)	13,801 (2.5)
**AKI or need for RRT – n (%)**	590 (3.4)	3,203 (4.1)	2,476 (3.6)	4,168 (3.4)	10,437 (3.6)	301 (2.0)	1,347 (2.2)	1,205 (2.4)	2,756 (2.0)	5,609 (2.1)	891 (2.7)	4,550 (3.3)	3,681 (3.1)	6,924 (2.7)	16,046 (2.9)
**Cardiac resuscitation– n (%)**	200 (1.2)	1,018 (1.3)	840 (1.2)	1,199 (1.0)	3,257 (1.1)	188 (1.2)	788 (1.3)	575 (1.2)	1,012 (0.7)	2,563 (1.0)	388 (1.2)	1,806 (1.3)	1,415 (1.2)	2,211 (0.9)	5,820 (1.1)
**Mechanical ventilation – n (%)**	4,825 (27.9)	22,507 (28.6)	16,400 (23.8)	20,294 (16.5)	64,026 (22.2)	4,440 (29.2)	19,767 (32.5)	13,510 (27.4)	21,585 (15.8)	59,302 (22.7)	9,265 (28.5)	42,274 (30.3)	29,910 (25.3)	41,879 (16.1)	123,328 (22.4)
**Median duration of ventilation – h (IQR)**	98 (338)	128 (362)	118 (332.5)	63 (231)	97 (315)	69 (301)	96 (326)	86 (289)	44 (157)	66 (262)					79 (290)
**In Hospital Death – n (%)**	1,203 (7.0)	7,657 (9.7)	9,377 (13.6)	27,581 (22.4)	45,818 (15.9)	1,099 (7.2)	6,166 (10.2)	7,452 (15.1)	37,320 (27.4)	52,037 (19.9)	2,302 (7.1)	13,823 (9.9)	16,829 (14.2)	64,901 (25.0)	97,855 (17.8)

**Mean length of hospital stay – days (SD)**	15.9 (24.0)	17.9 (24.4)	16.6 (22.2)	13.6 (16.9)	15.6 (21.0)	16.0 (23.4)	17.9 (23.7)	16.8 (21.5)	12.7 (14.9)	14.9 (19.2)					15.3 20.2)
**Mean charges per case – EUR (SD)**	12,464 (21,674)	13,536 (22,619)	11,816 (20,547)	8,564 (14,030	10,908 (18,869)	12,213 (19,875)	14,070 (21,921)	11,896 (19,207)	7,513 (11,498)	10,090 (16,691)					10,518 (17,868)

AKI indicates acute kidney injury; CABG, coronary artery bypass grafting; EA, endarterectomy; IQR, interquartile range; PCI, percutaneous coronary intervention; RRT, renal replacement therapy; SD, standard deviation
A dash - indicates no data due to data protection reasons.
